# Chitosan nanoplatform for the co-delivery of palbociclib and ultra-small magnesium nanoclusters: dual receptor targeting, therapy and imaging

**DOI:** 10.7150/ntno.94364

**Published:** 2024-02-12

**Authors:** Abhishesh Kumar Mehata, Virendra Singh, Vikas, Prachi Srivastava, Biplob Koch, Manoj Kumar, Madaswamy S. Muthu

**Affiliations:** 1 Department of Pharmaceutical Engineering and Technology, IIT (Banaras Hindu University), Varanasi-221005, UP, India.; 2 Cancer Biology Laboratory, Department of Zoology Institute of Science, (Banaras Hindu University), Varanasi-221005, UP, India.; 3 Nano2Micro Material Design Lab, Chemical Engineering and Technology, IIT BHU, Varanasi-221005, UP, India.

**Keywords:** chitosan nanoparticles, breast cancer, dual receptor targeting, optical and ultrasound/photoacoustic imaging, theranostics

## Abstract

Theranostic nanoparticles have gained significant attention in cancer diagnosis and therapy. In this study, estrone (ES) and folic acid (FA) functionalized single and dual receptor targeted theranostic chitosan nanoparticles were developed for breast cancer imaging and therapy. These nanoparticles (NPs) were loaded with palbociclib (PB) and ultra-small magnesium nanoclusters (UMN). The developed nontargeted theranostic NPs (PB-UMN-CS-NPs), estrogen receptor targeted theranostic NPs (PB-UMN-CS-ES-NPs), folate receptor targeted theranostic NPs (PB-UMN-CS-FA-NPs), and dual targeted theranostic NPs (PB-UMN-CS-ES-FA-NPs) have particle sizes of 178.4 ± 1.21 nm, 181.6± 1.35 nm, 185.1± 1.33 nm, and 198.2± 1.43 nm with surface charges of +19.02± 0.382 mV, +13.89±0.410 mV, +16.72±0.527 mV and +15.23±0.377 mV, respectively. Cytotoxicity studies on estrogen receptor (ER) and folate receptor (FR) expressing breast cancer cells revealed that dual-targeted theranostic NPs (PB-UMN-CS-FA-ES-NPs) were more effective, inhibiting cell growth by 54.17 and 42.23 times in MCF-7 and T-47D cells compared to free PB, respectively. Additionally, developed NPs were capable of inhibiting the cell cycle progression of MCF-7 cells from the G1 phase to the S phase more efficiently compared to free PB. Ultrasound and photoacoustic (USG/PA) imaging demonstrated that dual targeted theranostic NPs were capable of effectively reducing hypoxic tumor volume and significantly suppressing tumor vascularity compared to free PB, nontargeted, FR targeted and ER targeted NPs. Moreover, *in vivo* optical imaging demonstrated tumor specific accumulation of the dual-targeted theranostic NPs. Furthermore, *in vitro* hemocompatibility and histopathological studies confirmed the biocompatibility of developed nanoformulations.

## 1. Introduction

Breast cancer is the most prevalent form of aggressive cancer that primarily affects women. In the United States, breast carcinoma is the most common type of cancer and ranks as 2^nd^ foremost determinant of mortality in females. Annually, around 250,000 new subjects of mammary carcinoma are identified in the USA 1. Globally, breast tumors continue to represent a significant health concern for women, impacting approximately 5 % of women worldwide and up to 12.5% of women in high-income countries 2. Recent decades have witnessed substantial advancements in diverse cancer management approaches, such as spanning chemotherapy, photodynamic and photothermal therapies, radiation therapy, and the utilization of magnetic resonance imaging (MRI) 3.

The three primary markers of breast tumor are human epidermal growth factor receptor 2 (HER2), progesterone receptor (PR), and estrogen receptor (ER) that help in determining the course of treatment by indicating the tumor's aggressiveness, sensitivity to hormones, and suitability for targeted therapies. Breast tumors are commonly classified into triple-positive breast cancer (shows the presence of ER, PR, and HER2) and triple-negative breast cancer (shows the absence of all three receptors, ER. PR and HER2) 4-6. According to reports, around 83% of individuals with breast tumors have active hormonal receptors that can respond to hormonal therapy 7. ER are overexpressed and widely distributed within breast tumors. These ERs are commonly located in the nuclear (ERα and ERβ) and plasma membrane (mER) regions of breast tumor cells. In healthy breast cells, the presence of ER expression is below 10%; however, in breast tumor cells, the level of overexpression exceeds 80% 8. The overexpression of the folate receptor (FR) in breast cancer depends on the severity and stage of the breast cancer. It was noted that FRα was overexpressed up to 86% in triple negative breast cancer 9.

Folic acid (FA) is a water-soluble dietary component essential for the growth of healthy cells and for fulfilling cellular nutritional demands by participating in DNA replication, amino acid conversion, and red blood cell (RBC) generation 10. The increased demand for FA in tumors, linked to accelerated cell division rates, prompts compensatory measures to meet the elevated folate needs essential for rapid cell proliferation 11. Several customized delivery techniques that have previously shown success involve utilizing NPs surfaces attached to FA. Employing FA as a targeting mechanism can be a potent strategy for improving intracellular delivery (internalization) while mitigating the risk of unintended distribution and damage to non-specific areas 12. The estrone (ES) has a preferentially binding affinity towards ER and is widely used for the formulation of targeted NPs for ER positive breast cancer 13. In research, Kurmi and colleagues fabricated dual cancer-targeted NPs utilizing ES-functionalized chitosan (CS) and doxorubicin. This approach was explored for its potential anticancer effects specifically targeted at MCF-7 cells 14. Recently, Tang *et al,* developed PEGylated liposomes with ES modifications to their surface to enable the concurrent administration of paclitaxel and epirubicin to rats bearing ER overexpressed breast cancers. The inclusion of ES on the surface of the liposomes resulted in increased cellular internalization and improved accumulation of the targeted NPs within MCF-7 cells and a mouse model harboring MCF-7-derived tumors, respectively 15.

Palbociclib (PB) is an anticancer agent approved for metastatic breast cancer, which works by targeting cyclin-dependent kinases CDK4 and CDK6. PB binds to the ATP pockets of CDK4 and CDK6 and inhibits their activity. However, the use of PB has been associated with side effects such as neutropenia, rash, itching or hives, and tiredness 16. Safety concerns regarding off-target delivery raise issues for fetal development, liver function, and vital organs, while PB interaction with the P-gp efflux pump contributes to chemotherapy resistance in tumor cells 17. Hence, developing a drug delivery system to mitigate these harmful effects and deliver PB to the targeted site is highly desirable. Additionally, incorporating the P-gp efflux pump inhibitor, such as D-α-tocopheryl polyethylene glycol 1000 succinate (TPGS), prevents drug resistance towards cancer cells by preventing the P-gp ATPase from hydrolyzing ATP by blocking the ATP binding sites 18.

Our bodies naturally contain significant amounts of magnesium (Mg), an alkaline earth metal that plays a crucial role as a metabolite and catalyst in various metabolic processes. When it transforms into the Mg^2+^ ion, the body readily absorbs it without causing any adverse effects on cytotoxicity or accumulation 19. Given these biological characteristics, metallic magnesium becomes an essential component for developing fluorescent nanoclusters with a broader *in vivo* application. In this research, we have used ultra-small magnesium nanoclusters (UMN) as an *in vivo* imaging agent for the development of theranostic NPs, due to their excellent biocompatibility, biodegradable, and nontoxic nature. UMN has a tendency to aggregate upon storage; however, with mild agitation, it gets redispersed 20.

Theranostic nanomedicine combines diagnostic and therapeutic capabilities in a single NPs platform. Additionally, the therapeutic efficacy of a single-receptor-targeting nanomedicine is limited due to the heterogeneity and complexity of breast tumors. The concept of dual-receptor-targeted nanomedicine is emerging as a promising approach to enhance breast cancer therapies. Further, reduced vascularization in hypoxic tumors hinders the effective penetration of anti-tumor drugs 21. Numerous dual-receptor targeted drug delivery systems have been designed to enhance targeting accuracy toward tumor sites by combining two targeting ligands. Examples include transferrin and folate 22, Arginylglycylaspartic acid (RGD) and transferrin 23, hyaluronic acid and folate 24, hyaluronic acid and transferrin 25. Breast cancer therapy is markedly impacted by tumor hypoxia, characterized by low oxygen levels, resulting in a threefold higher reported therapeutic resistance compared to normoxic tumors 26.

Hence, understanding the treatment needs for addressing advanced stage breast tumors, a targeted theranostic nanomedicine that facilitates the precise imaging of the breast tumor and delivers anticancer agents to tumors, becomes an attractive option. To achieve targeted imaging and therapy for advanced-stage breast cancer, we have developed theranostic NPs with targeting capabilities for the FR, ER, and dual receptors (FR and ER), and loaded them with PB and UMN. We have conducted a comprehensive set of physicochemical and *in vitro* characterizations of NPs. We have investigated the qualitative cellular uptake of these theranostic NPs in breast cancer cell lines (MCF-7 and T-47D cells) that overexpressed with ER and FR. Furthermore, we evaluated the *in vitro* cellular cytotoxicity of PB-loaded NPs, including nontargeted, ER-targeted, FR-targeted, and dual-targeted NPs, in MCF-7 and T-47D cells to determine their IC_50_ values. In addition, we performed a histopathological examination of vital organs such as the brain, lungs, liver, kidney, and spleen in normal rats to assess potential organ toxicity resulting from nanoformulation treatments, respectively. Moreover, we evaluated the anticancer effects of the NPs in Sprague Dawley (SD) rats with 7,12-Dimethylbenz[a]anthracene (DMBA) induced hypoxic breast tumor. Simultaneously, we employed ultrasound and photoacoustic imaging systems to visualize tumor size, hypoxic tumor region and tumor vascularity in rats. Furthermore, *in vivo* tumor targeting and distribution of the NPs in rats were quantified using optical imaging.

## 2. Materials and Methods

### 2.1. Materials

Sun Pharmaceutical Industries Ltd, Gurugram, India, provided complimentary palbociclib (PB) samples. Chitosan (CS) with a molecular weight of approximately 1.5 kDa and a degree of deacetylation greater than 90%, as well as Estrone (ES), Folic acid (FA), succinic anhydride (SA), Sodium-tripolyphosphate (Na-TPP), 4-(Dimethylamino)pyridine (DMAP), N-Hydroxy succinimide (NHS), triethylamine (TEA), and 1-Ethyl-3-(3-dimethylaminopropyl)carbodiimide (EDC), were sourced from SRL, India. Magnesium chloride was supplied by Merck India. Estrone succinate (ES-COOH) 27, chitosan-estrone (CS-ES) 14, 28, and chitosan-folate (CS-FA) 29 were prepared by previously reported methods. MCF-7 and T-47D human breast cell lines were acquired from NCCS Pune, India. Genetix Biotech Asia Pvt. Ltd, supplied chemicals and nutrients for cell work. Eppendorf provided 96-well plates and T-25 cell culture flasks. Invitrogen Thermo Fisher Scientific supplied 3-(4,5-Dimethylthiazol-2-yl)-2,5-Diphenyltetrazolium Bromide (MTT). All other chemicals used in the research were of analytical grade.

### 2.2. Methods

#### 2.2.1. Preparation of UMN

Ultra-small magnesium nanocluster (UMN) as an imaging agent was prepared as per the method reported by Srivastava et al 20. UMN as an imaging agent, was prepared as per the reported method. Briefly, all the glassware was cleansed with aqua regia (HCl(3): HNO_3_ (1)) and then rinsed two to three times with distilled water and ethanol. MgCl_2_ salt solution (10 mM, 10 mL) and BSA solution (450 mg/mL, 1 mL) were used to synthesize BSA-templated magnesium nanoclusters. L-ascorbic acid (35 mg/mL, 3 mL) was added after mixing both solutions for 5 min (55°C, 900 rpm). The solution was then maintained for 2 hr at 55° C with constant vigorous agitation. After 2 hr of reaction, the colourless solution became pale yellow. The solution was then incubated for 15 hr at 55° C. The colour of the solution changed from light to pale yellow, indicating the completion of the reaction. The nanoclusters were centrifuged (15,000 rpm for 10 min) to remove larger particles. Further, UMN suspension was freeze dried and stored at 4°C. The prepared UMN was characterized for fluorescent intensity and transmission electron microscopy.

#### 2.2.2. Formulation of theranostic NPs

Theranostic NPs were developed by using UMN as an imaging agent and PB as a therapeutic agent. The co-loading of both components in the CS-NPs was achieved by modified emulsion solvent evaporation followed by an ionic crosslinking technique 30. In brief, a solution of the 30 mg chitosan was prepared in 0.2 % v/v of the acetic acid, and 2 mg of the UMN was transferred to it, and vortexed to get a homogeneous solution. The solution pH 6.0 was adjusted by using dil. NaOH and added 20 mg of the TPGS (surfactant). Separately, 3 mg of the PB was dissolved in 1 mL of chloroform. Both polymeric solution and drug solution were mixed and the emulsion was prepared by using an ultrasonic probe sonicator (Hielscher UP200H, Germany). The formed emulsion was magnetically stirred for 4 hr. After evaporation of the chloroform, a dropwise addition of 1 mL of sodium TPP (1 mg/mL) solution was added for ionic crosslinking of NPs suspension. The developed NPs were subjected to centrifugation at 3000 rpm for 10 min to remove larger NPs. Additionally, after separation of the larger NPs, desired NPs were collected from the NPs suspension by centrifugation at 14000 rpm for 10 min. The obtained pellets were washed with distilled water and suspended in phosphate buffer saline (pH 7.4).

For the targeted delivery of the NPs, they were prepared as per the above-mentioned methods, with the replacement of 10 mg of CS with 10 mg CS-ES (for ER targeting), 10 mg of CS-FA (for folate receptor targeting) and 10 of each CS-ES and CS-FA (for dual receptor targeting). **Table 1** displays the composition of the different theranostic NPs formulations.

The formulation of chitosan NPs using sodium TPP involves a complex process that leverages the interaction between the positively charged amino groups of chitosan and the negatively charged phosphate groups of sodium TPP. A spontaneous electrostatic interaction between chitosan and sodium TPP leads to the formation of polyelectrolyte complexes. These complexes act as crosslinks between chitosan chains, resulting in the formation of NPs. Sodium TPP serves as a crosslinking agent, facilitating the formation of a three-dimensional network within the chitosan solution. As the crosslinking progresses, the NPs stabilize due to the electrostatic interactions and physical entanglement between the chitosan chains and sodium TPP molecules. Since, chitosan was preconjugated with ES or FA, targeting moiety will be assembled on the surface of targeted NPs.

### 2.3. Characterization of nanoparticles

#### 2.3.1. Particle size, zeta potential and polydispersity index

The developed NPs were subjected to analysis by using a Zetasizer (Nano ZS90, Malvern Instruments) to obtain the average particle size, polydispersity, and zeta potential. The values presented are the means of three measurements.

#### 2.3.2. Entrapment efficiency

The entrapment efficiency (% EE) of PB within the nanoformulations were analyzed by utilizing reverse phase validated HPLC (SIMADZU LC-20AR, Japan) method 31. In order to rupture the NPs, 200 µL of the NPs dispersion was dried and then mixed with 1 mL of methanol and bath sonication was done for 1 hr. Upon an appropriate dilution in the mobile phase, it passed through a 0.22 µm nylon filter and was then subjected to HPLC analysis. The standard calibration curve for PB exhibited linearity with an R^2^ value of 0.998, covering a concentration range of 10 to 60 ng/mL. To quantify PB, we employed a validated HPLC analytical method. This method utilized a mobile phase composed of acetonitrile (30%) and methanol (70%). The HPLC parameters were a flow rate of 1 mL/min, an injection volume of 100 μl, a retention time of 4.8 min, and a PDA detector with a maximum wavelength set at 355 nm (λmax). The HPLC system had a Shimadzu Shim-pack C18 column, which played a critical role in separating and quantifying PB.

The amount of UMN entrapped in the chitosan NPs were determined using an indirect method involving a multimode microplate reader in fluorescent mode. After completing the sample preparation process, measurements were carried out in fluorescence mode with an excitation wavelength of 469 nm and an emission wavelength of 543 nm. The resulting standard curve showed linearity within the concentration range of 10-100 µg/mL, with an R² value of 0.999.

% EE of PB or UMN were determined by the following formulae:



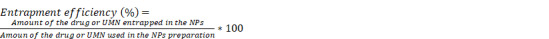



#### 2.3.3. High resolution scanning electron microscopy (HR-SEM)

Morphology of the prepared PB-UMN-CS-NPs, PB-UMN-CS-FA-NPs, PB-UMN-CS-ES-NPs and PB-UMN-CS-FA-ES-NPs were obtained by HR-SEM (Nova Nano SEM 450, FEI USA). The PB-UMN-CS-NPs, PB-UMN-CS-FA-NPs, PB-UMN-CS-ES-NPs and PB-UMN-CS-FA-ES-NPs suspensions were diluted tenfold with purified water, NPs suspension were placed onto a coverslip separately, ensuring even distribution. The coverslip was then placed in a vacuum drier for overnight. Once the dried thin films were prepared, a layer of carbon was applied to them, and microscopic photographs were taken by HR-SEM 32*.*

#### 2.3.4. Transmission electron microscopy (TEM)

PB-UMN-CS-NPs, PB-UMN-CS-FA-NPs, PB-UMN-CS-ES-NPs and PB-UMN-CS-FA-ES-NPs images were taken by utilizing TEM (FEI Tecnai G2 F20 X-TWIN). The suspensions of nanoformulations were tenfold diluted in purified water before being deposited onto a separate TEM grid with a 400-mesh. The TEM grid, with the dried NPs cast on it, was then subjected to vacuum drying. Subsequently, TEM was used to capture images of the samples 33.

#### 2.3.5. Atomic force microscopy (AFM)

Furthermore, using AFM (NTEGRA Prima, Netherlands), pictures of the NPs in two and three dimensions were taken. Following a tenfold dilution with purified water, a droplet of the NPs samples was cast onto individual glass slides and evenly spread to create thin films. Additionally, samples cast as films underwent low-pressure vacuum drying in a vacuum drier. The images were then taken and analyzed using AFM image analysis software (Nova Px from NT-MDT, Netherlands) 29.

#### 2.3.6. Surface analysis by XPS

Surface elemental composition of the PB-UMN-CS-NPs, PB-UMN-CS-FA-NPs, PB-UMN-CS-ES-NPs and PB-UMN-CS-FA-ES-NPs were analyzed by X-ray photoelectron spectroscopy (XPS, K-Alpha, Thermo Fisher Scientific Inc.). A dried thin film of the NPs was scanned for 10-800 eV of binding energy to detect C, N, O and Mg content in the samples 34.

#### 2.3.7. ES/FA content

PB-UMN-CS-ES-NPs and PB-UMN-CS-FA-ES-NPs were processed for the calculation of their ES content on the NPs surface was performed by using a multimode microplate reader. Briefly, 0.3 mL of NPs were dried and then mixed with a solution of DMSO to DCM in a 4:1 ratio. After 4 hr of vortexing and subsequent centrifugation, the processed samples supernatant was filtered through a 0.22 µm filter and subjected to analysis at 296 nm 35. The amount of ES present in the PB-UMN-CS-ES-NPs and PB-UMN-CS-FA-ES-NPs was determined by the given formula.



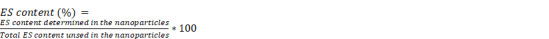



Similarly, for determining FA content in the PB-UMN-CS-FA-NPs and PB-UMN-CS-FA-ES-NPs, samples were processed as per the above method and the absorbance of the sample was recorded at 364 nm. The % FA content was estimated by using the following method 36.



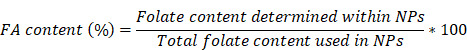



#### 2.3.8. X-ray diffraction study (XRD)

The pure drug, excipients and lyophilized NPs were scanned by Rigaku MiniFlex X-ray diffractometer in the range of 5-80° (2θ) by applying a voltage of 40kV with 5°/min scan rate. The XRD analysis will be helpful in the identification of the physical state of drug distribution within the NPs 37.

### 2.4. In vitro studies

#### 2.4.1. In vitro drug release

The dialysis bag diffusion techniques were used for the *in vitro* release of PB from NPs. The NPs suspension containing 300 µg of the PB was kept inside the dialysis bag (1000 Da) and sealed on both ends. Further, an *in vitro* release study was carried out at 37±0.5°C in a beaker containing 100 mL of PBS 7.4 pH. The sealed dialysis tube was immersed, and continuous shaking of the setup was maintained at 100 rpm. At each predetermined sampling time point, 1 mL of the sample was withdrawn and replaced with 1 mL of fresh buffer. Additionally, free PB was also tested for *in vitro* release. Similarly, free drug and developed NPs *in vitro* drug release were evaluated at pH 6.5 PBS and pH 4.5 acetate buffer. Samples were diluted, filtered and collected in HPLC vials for analysis by using a validated RP-HPLC analytical method.

#### 2.4.2. Evaluation of hemocompatibility

The evaluation of biocompatibility between the NPs and blood was carried out to assess their impact on red blood cells. A 10 mL blood sample was taken in a heparinized tube. Red blood cells (RBCs) were then isolated after centrifuging at 3000 rpm. The RBC that settled at the bottom of the tube underwent 2-3 washes with a normal saline solution before being suspended in the same solution. Additionally, 0.9 mL of RBCs suspension and 0.1 mL of different NPs (i.e., PB, PB-UMN-CS-NPs, PB-UMN-CS-FA-NPs, PB-UMN-CS-ES-NPs and PB-UMN-CS-FA-ES-NPs) were incubated together.

RBCs suspensions in distilled water served as the positive control, while RBCs suspensions in saline were used as the negative control. These samples were kept at 37°C with gentle shaking for 1 hr. When RBCs take up water and subsequently swell and burst, distilled water causes 100% hemolysis. Following the incubation period, smears were prepared, and Leishman stain was applied to individual glass slides with a drop of the specimens. After staining, a bright microscope was employed to capture images of the stained RBCs. Furthermore, the incubated specimens underwent a 5 min centrifugation at 3000 rpm. The supernatants from these samples were collected, and their UV absorbance at 540 nm was measured utilizing a microplate reader. As per the ASTM E2524-22 standard, test materials below 5% of hemolysis are safer, whereas if hemolysis exceeds 5%, then test materials are hemolytic in nature and not suitable for *i.v.* administration 38.



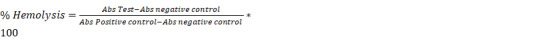



#### 2.4.3. In vitro physiological stability

The *in vitro* physiological stability of the developed NPs were evaluated in the plasma and serum of female rats. Briefly, equal volumes of the NPs suspension and plasma or serum were incubated at 37°C for 24 hr, separately. Following incubation, NPs were collected and evaluated for particle size, zeta potential and entrapment efficiency.

### 2.5. Maintenance of the cell lines

Dulbecco's Modified Eagle Medium (DMEM) supplemented with 10% Fetal Bovine Serum (FBS) and the antibiotic solution Penicillin-Streptomycin was employed for the culture of T-47D and MCF-7 breast cancer cells. Throughout the study, a 5% CO_2_ environment was maintained, and the T-47D and MCF-7 cells were cultured in a humidified CO_2_ incubator.

#### 2.5.1. Cytotoxicity assay

The free PB, PB-UMN-CS-NPs, PB-UMN-CS-FA-NPs, PB-UMN-CS-ES-NPs and PB-UMN-CS-FA-ES-NPs cytotoxicity assay were performed in ER and FR overexpressed T-47D and MCF-7 cells. In brief, DMEM-seeded cells were cultured at a density of 1 x 10^4^ cells per well for 12 hr at 37°C in a CO_2_ humidified incubator with 5% CO_2_. After this 12 hr incubation period, the used medium was discarded, and the cells were incubated with various concentrations of free PB, PB-UMN-CS-NPs, PB-UMN-CS-FA-NPs, PB-UMN-CS-ES-NPs, and PB-UMN-CS-FA-ES-NPs (0.01 µg/mL, 0.1 µg/mL, 1 µg/mL, 10 µg/mL, 100 µg/mL), all diluted in DMEM medium. The cells were then cultured for an additional 24 hr. A volume of 100 μL of media containing MTT (5 mg/mL in PBS, pH 7.4) was added to each well of the plate after the removal of the drug containing media. The plate was subsequently incubated for 2 hr. The formazan crystals were not disturbed during this process, and the crystals were subsequently rinsed and dried for an additional 2 hr. Following 2 hr of washing and drying period, 100 µL of DMSO was added to each well. The optical densities of the samples were then measured at 570 nm using a microplate reader (BioRad Multiplate reader) 39. The determination of cellular viability was carried out utilizing the given formula.:



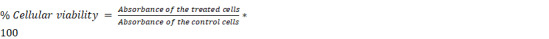



#### 2.5.2. Confocal laser scanning microscopy (cellular uptake)

The cellular internalization process of developed NPs, including free UMN, PB-UMN-CS-NPs, PB-UMN-CS-FA-NPs, PB-UMN-CS-ES-NPs, and PB-UMN-CS-FA-ES-NPs, were examined in the MCF-7 and T-47D cell lines using confocal microscopy (Wetzlar, Germany). Initially, a population of 1×10^5^ MCF-7 and T-47D cells was cultured for 24 hr on a coverslip placed within a 6-well cell culture plate, separately. Subsequently, the cells were treated with individual samples containing free UMN, PB-UMN-CS-NPs, PB-UMN-CS-FA-NPs, PB-UMN-CS-ES-NPs, and PB-UMN-CS-FA-ES-NPs, each at a concentration of 20 µg/mL (with respect to UMN), for 2 hr. Following this incubation, the cells underwent two rounds of cold phosphate-buffered saline (PBS) washing. After the incubation period, the cells were fixed using a 4% paraformaldehyde solution and then subjected to three additional washes with cold phosphate-buffered saline (PBS). To visualize the fixed cells nuclei, they were stained with propidium iodide (PI) during a 30-minute incubation. In experiments involving receptor blocking to study cellular uptake, the cells were pretreated with free ES and FA at a concentration of 2 mg/mL for 6 hr. This pretreatment was followed by treatment with PB-UMN-CS-FA-ES-NPs. The cellular monolayers were visualized using confocal microscopy 39.

#### 2.5.3. Cell cycle analysis

A study was conducted to analyze the cell cycle of developed nanoformulations, including free PB, PB-UMN-CS-NPs, PB-UMN-CS-FA-NPs, PB-UMN-CS-ES-NPs, and PB-UMN-CS-FA-ES-NPs, in MCF-7 cell lines 16. In brief, 1 x 10^5^ MCF-7 cells were cultured in 6-well plates using complete DMEM and allowed to adhere for 24 hr in a CO_2_ incubator. Subsequently, the MCF-7 cells were individually incubated with free PB, PB-UMN-CS-NPs, PB-UMN-CS-FA-NPs, PB-UMN-CS-ES-NPs, and PB-UMN-CS-FA-NPs. Following the treatment, the cells were collected using a chilled PBS containing 1 mM EDTA. The cells were fixed with 70% ethyl alcohol and then incubated at -20 °C for 12 hr. Subsequently, the cells were treated with a PI-RNAse solution containing 1 mg/mL PI, 0.1% V/V Triton X-100, and 10 mg/mL RNAse, and they were maintained at 37 °C for 30 min before being rinsed with chilled PBS. The cell cycle analysis was performed using a flow cytometer (CytoFLEX, Beckman Coulter, USA).

### 2.6. Histopathology study

Histopathological analysis was employed to assess the safety and toxicity of the developed nanoformulations after repeated dosing in rats (n=3). The groups included vehicle control (saline), drug control (free PB), folate receptor-targeted NPs (PB-UMN-CS-FA-NPs), ER-targeted NPs (PB-UMN-CS-ES-NPs), and dual-targeted NPs (PB-UMN-CS-FAES-NPs). These nanoformulations were administered intravenously at doses of 5.91 mg/kg (equivalent to PB), with three days between each dose. On the fifteenth day, all groups of animals were euthanized, and their vital organs were carefully excised and washed with PBS before being mounted in a cryostat. Tissue specimens were then sliced into sections with a thickness of 5 μm using a Leica CM1950 cryomicrotome. Subsequently, the sections were stained with hematoxylin and eosin (H & E) dye for histological examination. The pictures of all specimens were collected by using a light microscope.

### 2.7*. In vivo* anti-tumor activity

#### 2.7.1. Animals

Female SD rats, with a weight of approximately 200 g and an age range of 60-70 days, were housed in a standard environment at ambient temperature and provided with a standard diet. The Institutional Animal Ethics Committee (IAEC) at the Indian Institute of Technology (Banaras Hindu University), Varanasi, India, approved all *in vivo* animal experiments (IAEC Approval Number: IIT(BHU)/IAEC/2022/047) and all experiments were conducted in accordance with the National Research Council's Guide for the Care and Use of Laboratory Animals.

#### 2.7.2. Tumor induction

Breast tumors in the SD rats were induced by using DMBA at a dose of 25 mg/kg body weight. DMBA was dissolved in the almond oil and injected into the rat's breast pad (left or right). At the beginning of the 8-week DMBA administration, all rats were assessed for the development of tumors. The mammary pad was palpated regularly during the screening process to detect the presence of any suspicious masses. After approximately 75 days of DMBA administration, the tumor size became stable 40-42. The rats were regularly palpitated once the tumor diameter became above 6 mm and were chosen for the *in vivo* experiments. Reports have demonstrated that DMBA induced breast tumors were found to have overexpressed ER and PR 43. Additionally, a cell line extracted from DMBA-induced breast cancers displays characteristics similar to those of the MCF-7 cell line 44.

#### 2.7.3. Grading of breast tumor

Breast tumors developed in rats after administration of DMBA were removed and cleaned with PBS. A 5 µm thick section was prepared from the tumor paraffin block using a microtome. The globally accepted standard method for distinguishing tumors from healthy tissue is Hematoxylin and Eosin (H & E) staining. In accordance with established procedures, an H & E stain was applied to the obtained tumor section. Healthy rat breast was also extracted and stained with H&E to compare with the breast tumor.

#### 2.7.4. In vivo breast tumor imaging by USG/PA

The *in vivo* anticancer investigation was conducted on female SD rats with induced breast tumors. Six groups, each consisting of three rats with breast tumors, were randomly assigned for the study. Before receiving NPs therapy, all rats with breast tumors underwent ultrasound and photoacoustic (USG/PA) imaging system scans using a 40-MHz ultrasound array transducer (Vevo LAZR_X, Toronto, Canada) 45, 46. Various imaging modes were employed to examine the mammary tumors. Each animal received an equivalent dose of 5.91 mg/kg of PB, either the free drug or NPs. Group I, serves as disease control (no drug treatment). In Group II, rats received *i.v.* injections of pure PB suspended in sterilized phosphate buffer saline. Group III rats were injected with PB-UMN-CS-NPs. Group IV was injected with PB-UMN-CS-FA-NPs. Group V rats were given PB-UMN-CS-ES-NPs. Finally, Group VI rats underwent treatment with PB-UMN-CS-FA-ES-NPs. Imaging at 0 days refers to imaging of rats prior to administration of free drug or formulations. After 2, 4, and 6 days following the treatments, USG/PA imaging of the rats was performed. Image processing was performed by the Vevo LAB software for each dataset 47.

#### 2.7.5. In vivo fluorescent imaging of the breast tumor

The targeted delivery of the theranostic NPs to the breast cancer bearing rat after *i.v.* administration of the developed theranostic NPs and free UMN was investigated by using the Photon IMAGER-Optima system (Biospace Lab). The *in vivo* fluorescence produced after administration of the free UMN (control) and PB-UMN-CS-NPs, PB-UMN-CS-FA-NPs, PB-UMN-CS-ES-NPs and PB-UMN-CS-FA-ES-NPs to the SD rats with breast tumor at an equivalent dose of 200 µg of UMN. Fluorescent data were collected at 469 nm excitation and 543 nm emission wavelengths at four different time points: 0.5, 2, 4, and 6 hr after injection. The obtained images were processed by using M3Vision software, and the ROI of circled breast tumors were calculated 34.

### 2.8. Statistical analysis

All the data of studies were demonstrated as a mean of three repetitive measurements with standard deviation. All the obtained data were statistically calculated by using GraphPad Prism 7.0. Statistical significance between groups was assessed using the one-way ANOVA and post-Tukey test. The following values were used to determine the statistical significance level: ns (P ≥0.05), * (P < 0.05), ** (P < 0.01), *** (P< 0.001), and **** (P< 0.0001).

## 3. Results and discussion

### 3.1. Characterization of UMN

**Figure 1:**
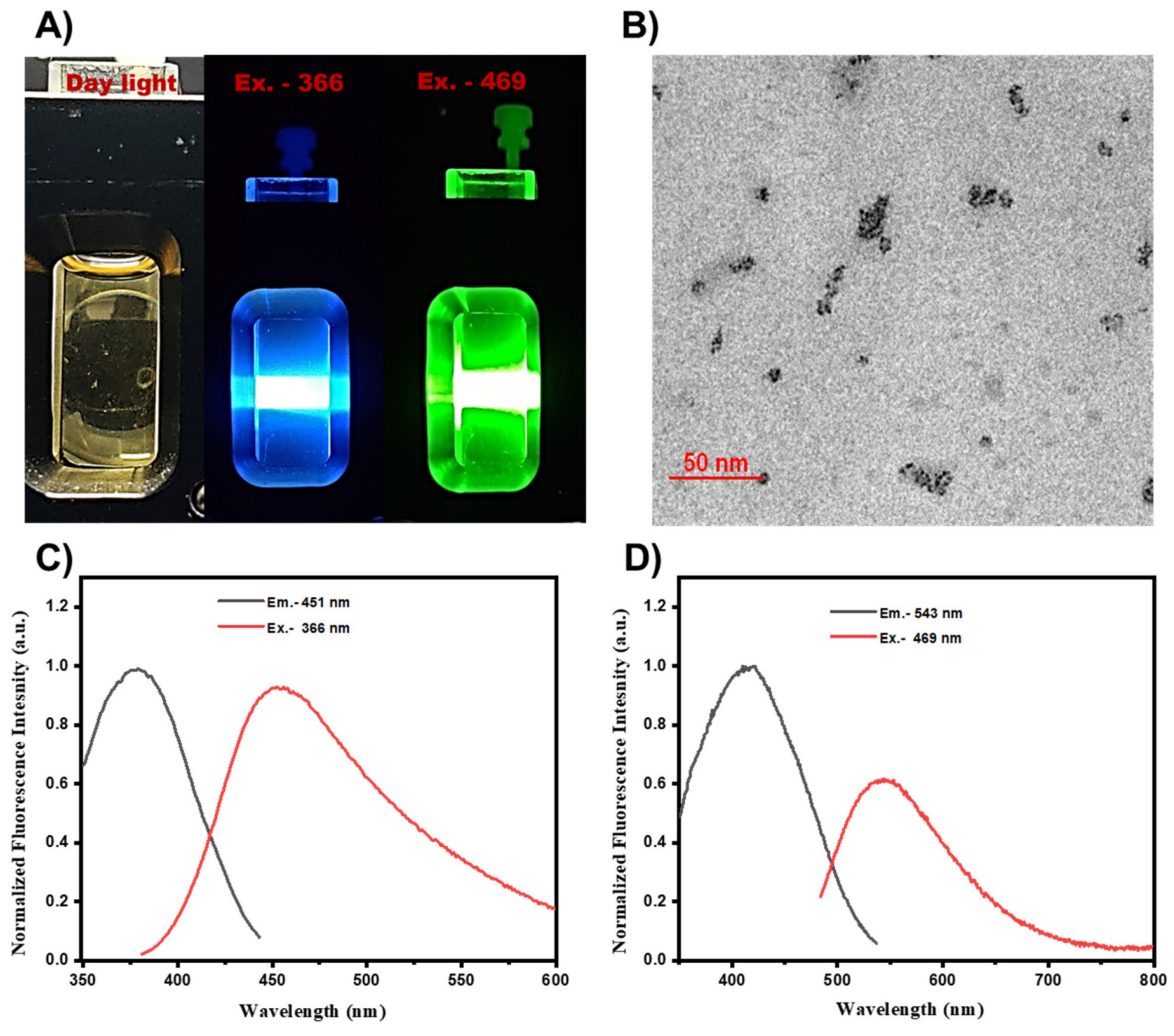
A) Fluorescent images of UMN at day light, Ex 366 nm (blue) and Ex 469 nm (green), B) TEM image of UMN, C) and D) UMN excitation and emission spectra.

Fluorescence spectra of the prepared UMN were collected by using a spectroflurophotometer. UMN suspension in water was subjected for the fluorescence scan, and images were captured. **Fig. 1A**. demonstrated the blue (excitation, 366 nm) and green (excitation, 469 nm) fluorescence of UMN. TEM analysis demonstrated the ultrasmall and uniform size of the UMN (**Fig. 1B**). Additionally, UMN aqueous suspension depicted 366 nm and 469 nm excitation spectra with 451 nm and 543 nm emission spectra, respectively (**Fig. 1C and 1D**). Hence, the developed UMN can be used as an imaging agent for *in vitro* and *in vivo* experiments.

### 3.2. Nanoparticles characterization

#### 3.2.1. Particle size, zeta potential and polydispersity index

The physicochemical evaluation of the developed nanoformulation has been presented in **Table 2**. The results demonstrated that prepared NPs had particle size in the range of 150.1 ± 1.83 nm to 198.2 ± 1.43 nm. The obtained data suggested that all NPs were below 200 nm. It was noted that blank CS-NPs had 150.1 nm and +20.62 mV particle size and zeta potential, respectively. Incorporation of PB inside NPs enhanced the particle size (178.4 nm) with little lowering in zeta potential (+19.02 mV). Additionally, in the case of FR targeted NPs and ER targeted NPs, incorporation of the CS-FA and CS-ES has significantly improved the particle size (185.1 nm and 181.6 nm) with lowered zeta potential (+16.72 mV and +13.89 mV), respectively. Additionally, dual-targeted NPs had 198.2 nm particle size and +15.23 mV zeta potential. The presence of ES and/or FA on the nanoformulation surfaces contributed to the increase in size. In contrast, the reduction of free NH_2_ in targeted NPs primarily accounted for the decrease in zeta potential.

#### 3.2.2. Entrapment efficiency evaluation

The amount of PB loaded in PB-UMN-CS-NPs, PB-UMN-CS-FA-NPs, PB-UMN-CS-ES-NPs and PB-UMN-CS-FA-ES-NPs were determined to be 74.15 ± 1.832 %, 75.92± 2.014 %, 76.82 ± 1.847 % and 73.04 ± 1.982 %, respectively (Table 2). Entrapment of PB was not much affected by the incorporation of the CS-ES and or CS-FA during the formulation of the targeted nanoformulation. Additionally, the amount of UMN entrapped in the PB-UMN-CS-NPs, PB-UMN-CS-FA-NPs, PB-UMN-CS-ES-NPs and PB-UMN-CS-FA-ES-NPs were found to be 58.24±1.08 %, 61.02±0.94 %, 57.31±1.07 % and 59.14±1.17 %, respectively.

#### 3.2.3. FE-SEM

**Figure 2:**
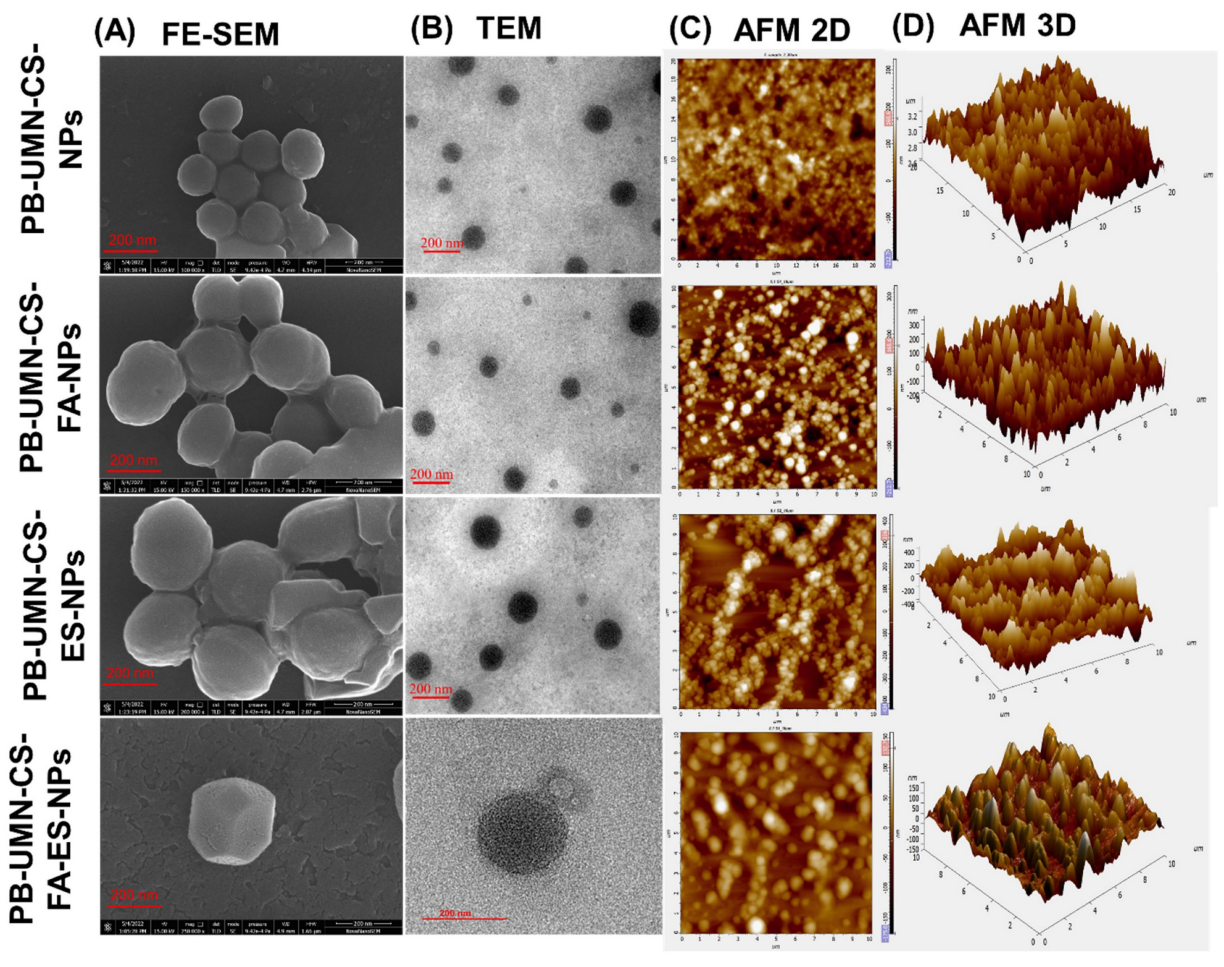
Microscopic images of PB-UMN-CS-NPs, PB-UMN-CS-FA-NPs, PB-UMN-CS-ES-NPs, and PB-UMN-CS-FA-ES-NPs by (A) FE-SEM, (B) TEM, (C) 2D AFM) and (D) 3D AFM.

The surface morphological images of the developed nanoformulation were taken by FE-SEM and presented in **Fig. 2A**. The microscopic images of the NPs depicted that developed formulations had round morphology with smooth surfaces. Additionally, from the images, it can also be confirmed that developed NPs had uniform particle size distribution 48.

#### 3.2.4. TEM

The images of the formulated NPs were captured using an electron beam with TEM. These techniques provide higher resolution photographs. TEM images of NPs at a scale of 200 nm are presented in **Fig. 2B**. Developed NPs appeared to exhibit a perfectly round shape in the images.

#### 3.2.5. AFM

The topography of the NPs in both two and three dimensions was examined using AFM. This technique yields high-quality images of the NPs without relying on optical methods. The two dimensional and three dimensional images of AFM, presented in **Fig. 2C** and **2D** revealed that all developed NPs were uniformly distributed and had round morphology.

#### 3.2.6. Surface chemistry by XPS

**Figure 3:**
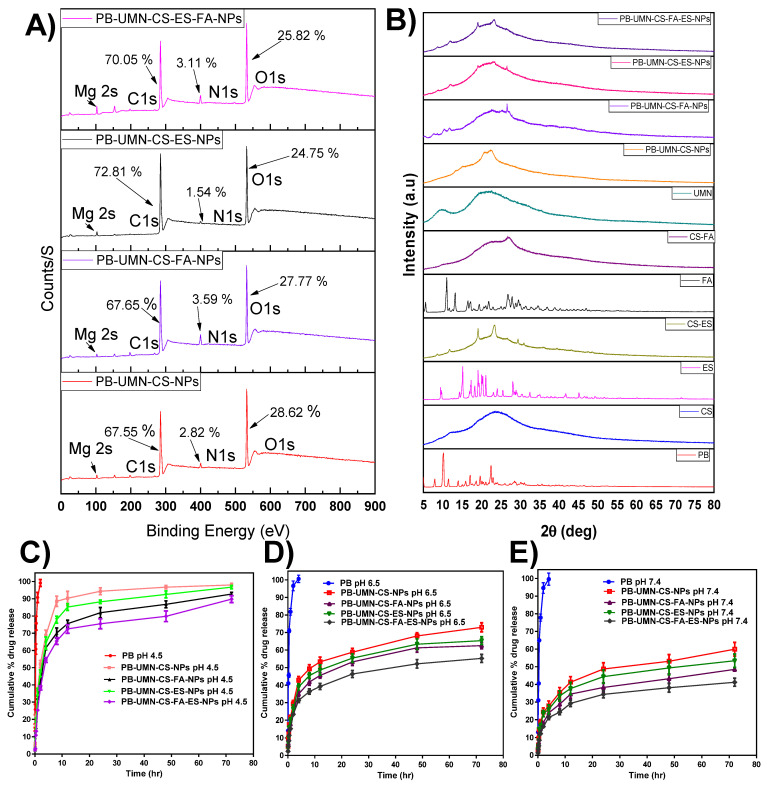
**(A)** XPS analysis of PB-UMN-CS-NPs, PB-UMN-CS-FA-NPs, PB-UMN-CS-ES-NPs and PB-UMN-CS-FA-ES-NPs by XPS, **(B)** XRD graph of PB, CS, ES, FA, UMN, CS-ES, CS-FA, PB-UMN-CS-NPs, PB-UMN-CS-FA-NPs, PB-UMN-CS-ES-NPs and PB-UMN-CS-FA-ES-NPs. *In vitro* PB release at **(C)** pH 4.5, **(D)** pH 6.5, and E) pH 7.4 from nanoformulations.

The NPs surface elemental composition was determined by using XPS. The developed NPs such as PB-UMN-CS-NPs, PB-UMN-CS-FA-NPs, PB-UMN-CS-ES-NPs and PB-UMN-CS-FA-ES-NPs elemental composition of C1s, N1s, O1s and Mg 2s were examined and presented in the **Fig. 3A**. The presence of the ES and/or FA on the surface of the NPs were identified based on the elemental composition of the formulation and comparison of the intensity of elemental signals of observed from developed formulations. The percentage of C1s, N1s and O1s in the PB-UMN-CS-NPs was observed to be 67.55 %, 2.82 % and 28.62 %, respectively. Similarly, PB-UMN-CS-FA-NPs, depicted of C1s, N1s and O1s as 67.65 %, 3.59 % and 27.77 %, respectively. PB-UMN-CS-ES-NPs demonstrated C1s, N1s and O1s signal with 72.81 %, 1.54 % and 24.75 %, respectively and PB-UMN-CS-FA-ES-NPs depicted C1s, N1s and O1s signal with an intensity of 70.05 %, 3.11 % and 25.82 %, respectively. The reduction % of nitrogen in the PB-UMN-CS-ES-NPs can be attributed to the prior conjugation of nitrogen atoms in the CS with ES. As a result, the nitrogen atoms on the surface of the PB-UMN-CS-ES-NPs were observed to be less abundant compared to the PB-UMN-CS-NPs. Additionally, the presence of a higher amount of the nitrogen signal in PB-UMN-CS-FA-NPs compared to PB-UMN-CS-ES-NPs confirmed the presence of FA on the surface of the PB-UMN-CS-FA-NPs. Further, the signal of the Mg 2s in all samples was ~1%, which confirmed the presence of UMN in the developed NPs.

#### 3.2.7. ES/FA content

The amount of ES present in PB-UMN-CS-ES-NPs and PB-UMN-CS-FA-ES-NPs were 78.10 ± 1.6 % and 74.85 ± 1.3 %, respectively. Similarly, the amount of FA present in PB-UMN-CS-FA-NPs and PB-UMN-CS-FA-ES-NPs were 73.24 ± 1.7 % and 71 ± 2.4 %, respectively.

#### 3.2.8. XRD Study

An XRD investigation enabled the assessment of drug phase identification in its nanoformulation. XRD analysis is a common technique used to track any alterations in the physical properties of a drug that may occur during the development of a nanoformulation. Most therapeutic agents occur in either a crystalline or amorphous state. In contrast to its crystalline state, the amorphous state offers improved solubility and enhanced bioavailability 49. XRD measurements of PB revealed multiple distinct and well-defined diffraction peaks at 2θ=7.92°, 10.14°, 11.52°, 13.94°, 15.96°, 16.95°,18.60°,19.68, 22.40°, and 22.68°. **Fig. 3B** indicates that pure PB can be observed in its crystalline state, confirming alignment with recently published findings 50. In PB-UMN-CS-NPs, PB-UMN-CS-FA-NPs, PB-UMN-CS-ES-NPs, and PB-UMN-CS-FA-ES-NPs, all of the PB peaks were absent, indicating that the drug had transitioned into an amorphous state due to the NPs formulations. This suggests that the resulting nanoformulation incorporated PB in the polymeric matrix. Since the amorphous state of the drug is more bioavailable than its the crystalline form, the prepared NPs may increase the bioavailability of PB. Additionally, it was observed that crystalline nature of the ES and FA has been reduced after conjugation of CS.

### 3.3. In vitro studies

#### 3.3.1. In vitro drug release studies

PB release profile from PB-UMN-CS-NPs, PB-UMN-CS-FA-NPs, PB-UMN-CS-ES-NPs and PB-UMN-CS-FA-ES-NPs at 7.4 pH, 6.5 pH and 4.5 pH have been demonstrated in **Fig. 3C-3E**. For simulating the physiological pH of a human, which is 7.4, *in vitro* release study was performed. Additionally, tumor microenvironment has a slightly acidic pH. In order to simulate tumor microenvironment *in vitro* release was performed at pH 4.5 and 6.5 51, 52.

The drug release pattern from PB-UMN-CS-NPs, PB-UMN-CS-FA-NPs, PB-UMN-CS-ES-NPs, and PB-UMN-CS-FA-ES-NPs depicted pH-dependent PB release characteristics. All nanoformulations exhibited rapid PB release properties at pH 4.5. This rapid release can be attributed to the protonation of the CS amino moiety, which enhances the dissolution of the amino group in water and facilitates faster PB release. Conversely, at pH 7.4, the protonation of CS was less pronounced, resulting in a compact NPs structure and gradual drug diffusion. In both pH media, free PB was released more rapidly due to the dialysis membrane acting as a regulatory barrier. The initial burst release from the NPs continued for up to 2 hr, likely due to drug release from the surface and the quicker diffusion of the drug near the surface of the NPs. Subsequently, slower drug diffusion from the NPs core and inner regions contributed to sustained PB release. PB is distributed throughout the polymer matrix of the NPs, which may necessitate more time for the drug to access the NPs surface. Moreover, the release pattern at pH 6.5 was faster than at 7.4 pH but slower than at 4.5 pH. Additionally, pH 4.5 (rapid release) can be advantageous for cancer treatment, given the acidic environment typically associated with tumors 53.

### 3.4. Hemolysis and hemocompatibility study

#### 3.4.1. Blood Smear

Nanomaterials fall within the size range of viruses, and immune-stimulating antigens have the potential to enhance the body's immune responses, induce inflammation, and influence hematological parameters 54. To assess the potential toxicities of PB, UMN, PB-UMN-CS-NPs, PB-UMN-CS-FA-NPs, PB-UMN-CS-ES-NPs, and PB-UMN-CS-FA-ES-NPs, we conducted hematological testing. In this research, deionized water, which induces 100% hemolysis, served as a positive control, while normal saline was used as a negative control (nonhemolytic). Blood samples were subjected to various formulation treatments and then incubated with Leishman stain to examine through a bright microscope (**Fig. S1A**). The collected images revealed that the shape and size of the blood cells were not significantly altered by exposure to PB, UMN, PB-UMN-CS-NPs, PB-UMN-CS-FA-NPs, PB-UMN-CS-ES-NPs, or PB-UMN-CS-FA-ES-NPs, and were comparable to the saline-treated samples.

#### 3.4.2. Hemolytic assay

Analyzing the percentage of hemolysis that happened after incubation with various nanoformulations enabled researchers to assess the hematological toxicity of PB, UMN, PB-UMN-CS-NPs, PB-UMN-CS-FA-NPs, PB-UMN-CS-ES-NPs, and PB-UMN-CS-FA-ES-NPs. Hemolysis % of PB, UMN, PB-UMN-CS-NPs, PB-UMN-CS-FA-NPs, PB-UMN-CS-ES-NPs and PB-UMN-CS-FA-ES-NPs were 2.825 ± 0.08 %, 1.813 ± 0.04 %, 1.201± 0.12 %, 1.310 ± 0.09, 1.152± 0.12 % and 1.382±0.10 %, respectively. The results indicated that PB, UMN, PB-UMN-CS-NPs, PB-UMN-CS-FA-NPs, PB-UMN-CS-ES-NPs, and PB-UMN-CS-FA-ES-NPs did not cause hemolysis in human blood (**Fig. S1B**).

### 3.5. In vitro physiological stability

The PS, ZP, and EE data of the NPs before and after incubation in plasma and serum demonstrated that the developed NPs were stable (**Fig. S2**). The variation in the PS, ZP, and EE before and after incubation did not show any significant changes (P> 0.05).

### 3.6. In vitro cytotoxicity assay

**Figure 4:**
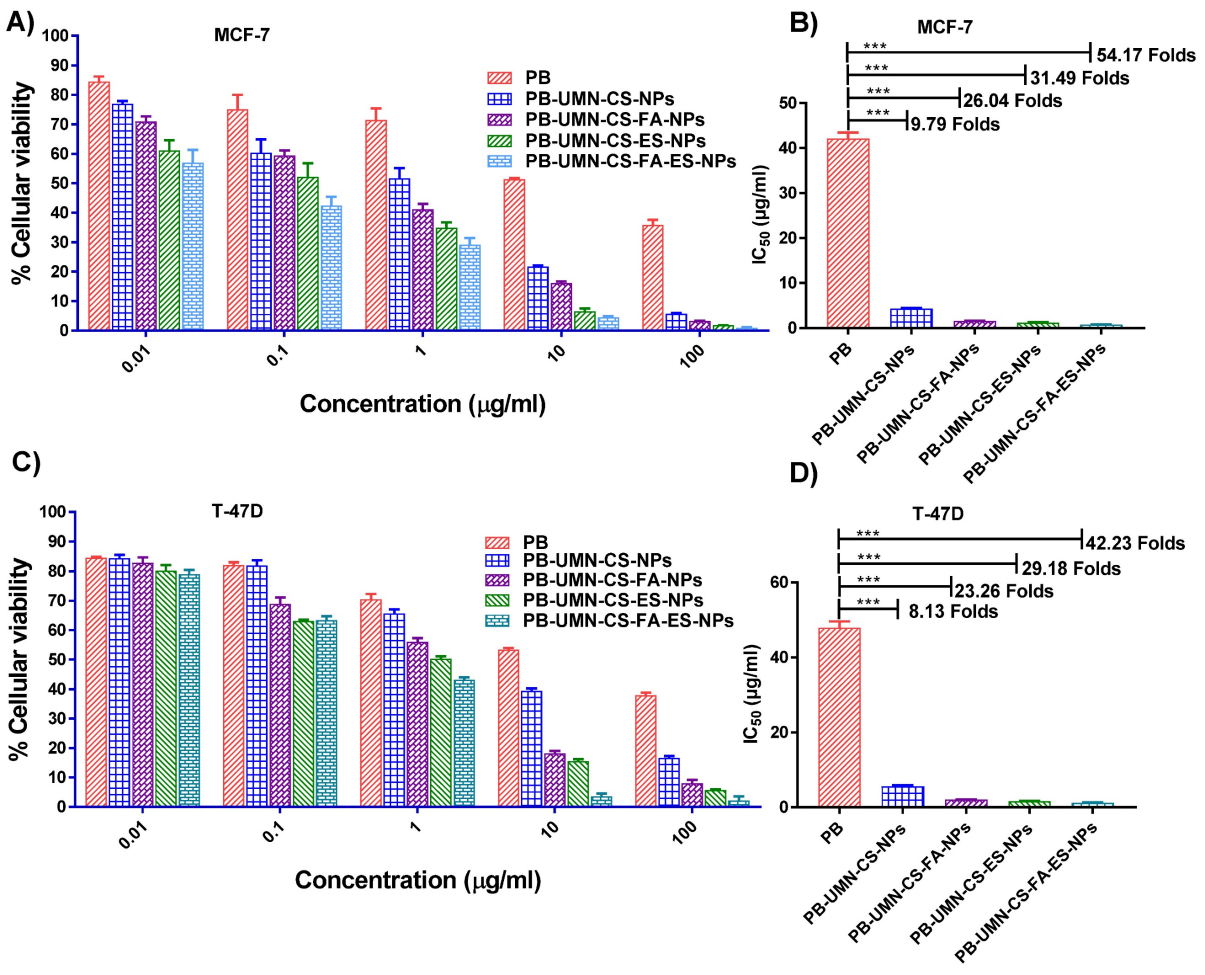
Cellular cytotoxicity assessment of the PB, PB-UMN-CS-NPs, PB-UMN-CS-FA-NPs, PB-UMN-CS-ES-NPs, and PB-UMN-CS-FA-ES-NPs on MCF-7 cells (A & B) and T-47D cells (C & D). *** (P< 0.001).

Based on the findings, pure PB does not significantly affect cell viability at lower concentrations. However, its efficacy in MCF-7 cells increased by approximately 9.79-fold when it was entrapped within nontargeted NPs. Furthermore, it was observed that receptor-mediated drug delivery using NPs was more efficient than passive drug delivery. Pure PB exhibited IC_50_ at ~40.63 µg/mL, while PB-UMN-CS-NPs, PB-UMN-CS-FA-NPs, PB-UMN-CS-ES-NPs, and PB-UMN-CS-FA-ES-NPs were found at ~4.15 µg/mL, ~1.29 µg/mL, ~1.56 µg/mL, and ~0.75 µg/mL respectively. **(Fig. 4 A & B)**. In T-47D cells the IC_50_ of PB was observed at ~48.15 µg/mL, while IC_50_ of PB-UMN-CS-NPs, PB-UMN-CS-FA-NPs, PB-UMN-CS-ES-NPs and PB-UMN-CS-FA-ES-NPs were observed at ~5.92 µg/mL, ~1.65 µg/mL, ~2.07 µg/mL, and ~1.14 µg/mL, respectively **(Fig. 4 C & D)**. The obtained IC_50_ value of the pure PB was in good agreement with the data reported in the literature 55. The presence of the receptor-targeting group in the developed nanoformulation significantly enhances the efficacy of PB, making it approximately 57 times more effective for cancer treatment. This substantial improvement is attributed to the overexpression of ER in MCF-7 and T-47D cells, which allows receptor-mediated targeted administration of PB to significantly enhance its anti-tumor effectiveness. Furthermore, it was observed that blank NPs had no significant cytotoxic effects on MCF-7 and T-47D cells, with cellular viabilities exceeding 95% and 96%, respectively. This demonstrates that blank nanoformulations were nontoxic to MCF-7 and T-47D cells.

In a recent study by Parsian *et al.,* magnetic dendrimers entrapped with PB were designed, and the researchers investigated the impact on cell viability in various mammary carcinoma cell lines. They found that PB-encapsulated magnetic dendrimers exhibited higher cytotoxicity against MCF-7 cells compared to MDA-MB-231 cells and SKBR3 cells. In MCF-7 cells, they observed up to a 30% reduction in cell viability 56.

### 3.7. Cellular uptake study

**Figure 5:**
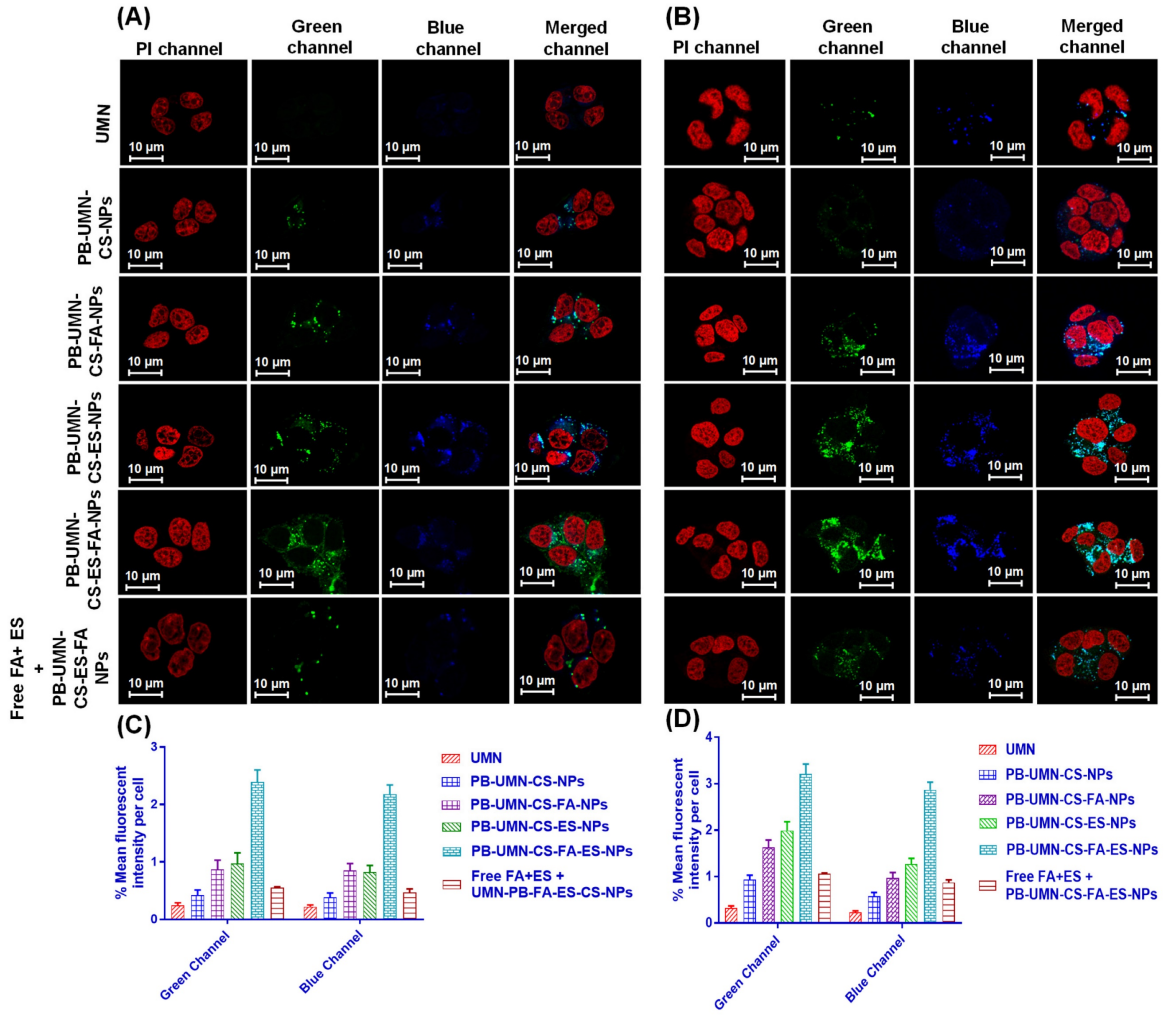
Cellular uptake of the free UMN (first row), PB-UMN-CS-NPs (second row), PB-UMN-CS-FA-NPs (third row), PB-UMN-CS-ES-NPs (fourth row), PB-UMN-CS-FA-ES-NPs (fifth row) and after pretreatment with FA+ ES (sixth row) in (A) T-47D cells and (B) MCF-7 cells. Scale bar showing 10 µm. % Mean fluorescent intensity per cell in (C) T-47D cells and (D) MCF-7 cells following cellular internalization of PB-UMN-CS-NPs, PB-UMN-CS-FA-NPs, PB-UMN-CS-ES-NPs and PB-UMN-CS-FA-ES-NPs.

A critical factor in predicting or assessing the therapeutic effectiveness of NPs is the cellular uptake of the developed NPs. In this current research, an active targeted drug delivery approach based on receptors was employed to enhance the cellular uptake of NPs in breast tumors that are both ER and FR. The incubation of the UMN, PB-UMN-CS-NPs, PB-UMN-CS-FA-NPs, PB-UMN-CS-ES-NPs, and PB-UMN-CS-FA-ES-NPs with MCF-7 cells and T-47D cells at 20 μg/mL of UMN depicted that PB-UMN-CS-NPs, PB-UMN-CS-FA-NPs, PB-UMN-CS-ES-NPs and PB-UMN-CS-FA-ES-NPs had increased cellular localization relative to the free UMN **(Fig. 5)**. Furthermore, in comparison to nontargeted NPs, both FR-targeted and ER-targeted, as well as dual-targeted NPs, showed significantly increased fluorescence. This suggests that the cellular localization of ES and/or FA-conjugated targeted NPs occurs through receptor-mediated cellular uptake. MCF-7 cells and T-47D cells have upregulated membrane estrogen receptors (mER) 57, 58.

Nontargeted NPs were taken up by cells through passive diffusion, involving the adherence of the nanoformulation to cancerous cells and its subsequent passive uptake into the cells. This passive uptake is facilitated by the leaky nature of blood vessels supplying the tumor, leading to increased penetration and retention effects by nontargeted NPs. Nontargeted nanoformulation was taken up by T-47D and MCF-7 cells (**Fig. 5A and 5B**). In contrast, targeted nanoformulation demonstrated improved cellular uptake due to the expression of ER and FR. Additionally, receptor inhibition with free ES and FA resulted in decreased cellular internalization of PB-UMN-CS-FA-ES-NPs, confirming ER/FR-mediated cellular internalization of NPs in MCF-7 and T-47D cells. Following the cellular uptake of UMN, PB-UMN-CS-NPs, PB-UMN-CS-FA-NPs, PB-UMN-CS-ES-NPs, and PB-UMN-CS-FA-ES-NPs, the percentage mean fluorescent intensity (green and blue channel) per cell in T-47D cells and MCF-7 cells was calculated using ImageJ software (**Fig. 5C and 5D**).

### 3.8. Cell cycle analysis

**Figure 6:**
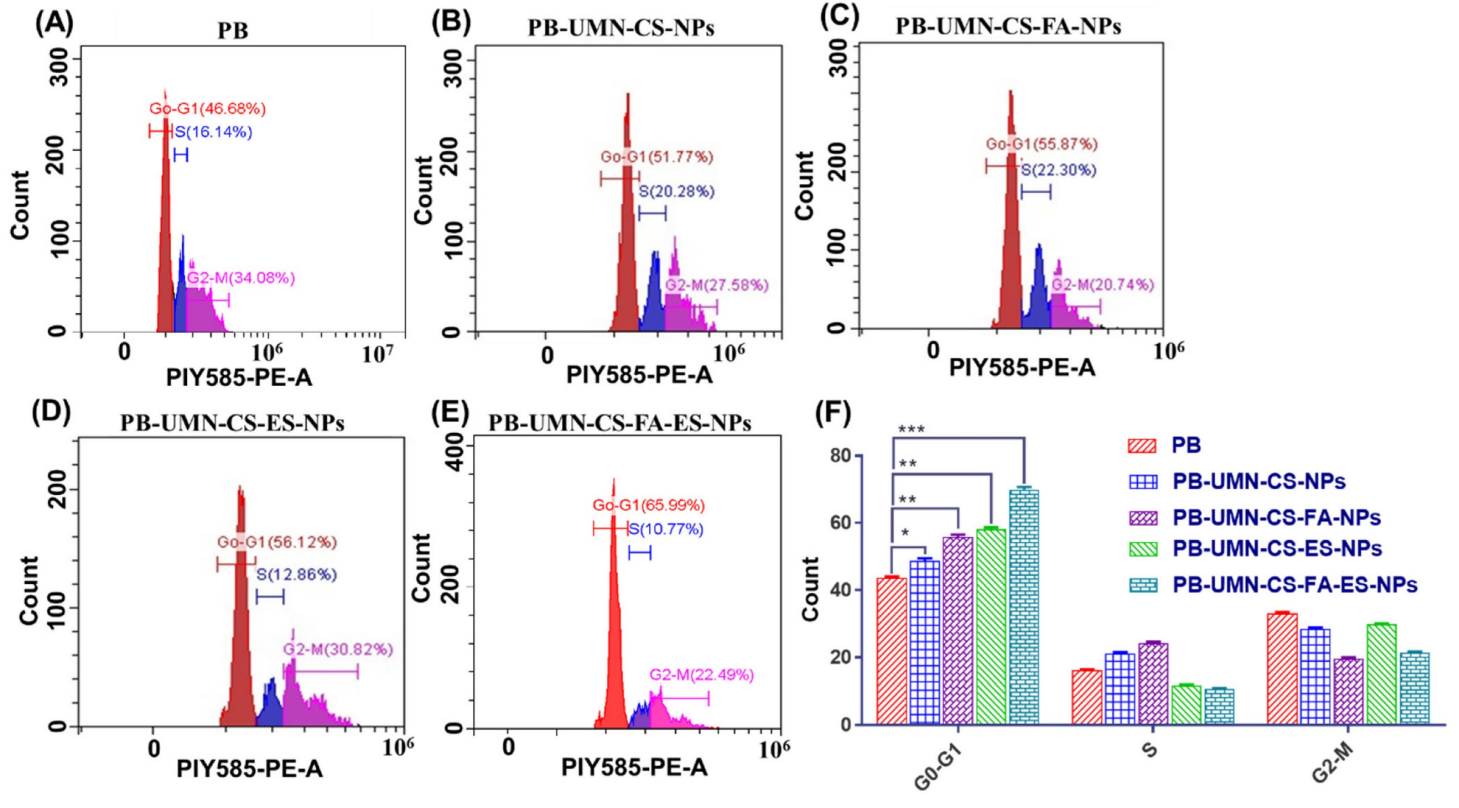
Cell cycle study of MCF-7 cells following incubation with A) free PB, B) PB-UMN-CS-NPs, C) PB-UMN-CS-FA-NPs, D) PB-UMN-CS-ES-NPs and E) PB-UMN-CS-FA-ES-NPs, F) Graphical presentation cell cycle study of free PB, PB-UMN-CS-NPs, PB-UMN-CS-FA-NPs, PB-UMN-CS-ES-NPs and PB-UMN-CS-FA-ES-NPs**.** * (P < 0.05), ** (P < 0.01), and *** (P< 0.001).

The use of flow cytometry analysis has confirmed the mechanism through which both the PB and PB-loaded NPs reduce the proliferation of MCF-7 cells. PB is a reversible inhibitor of CDK4/6. Its mechanism of action involves inhibiting the phosphorylation of retinoblastoma (Rb) protein, which, in turn, hinders the progression of the cell cycle from the G1 phase to the S phase. After treating MCF-7 cells with PB, PB-UMN-CS-NPs, PB-UMN-CS-FA-NPs, PB-UMN-CS-ES-NPs, and PB-UMN-CS-FA-ES-NPs, the cells were stained with PI and analyzed using a flow cytometer. The collected data were analyzed with Cytoflex software. The prepared formulations, PB-UMN-CS-NPs, PB-UMN-CS-FA-NPs, PB-UMN-CS-ES-NPs, and PB-UMN-CS-FA-ES-NPs, have significantly improved cell cycle arrest in the G1 phase, with increases of approximately 1.11-fold, 1.27-fold, 1.33-fold and 1.60-fold increases, respectively, relative to pure PB (**Fig. 6**). Cellular uptake and the MTT test results were in good agreement with the results of the cell cycle study.

### 3.9. Histopathology Study

**Figure 7:**
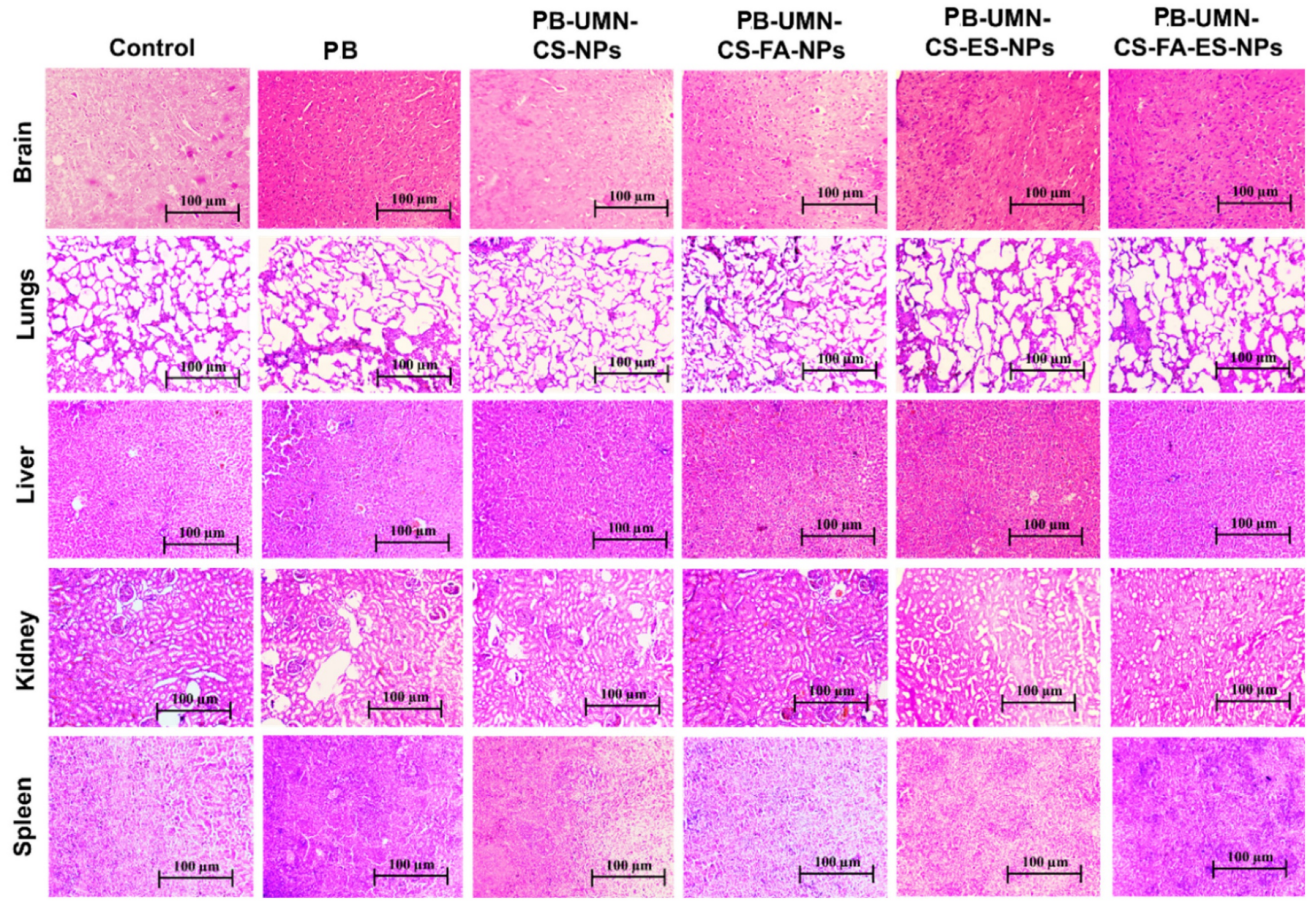
H & E-stained images following administration with saline (control), PB, PB-UMN-CS-NPs, PB-UMN-CS-FA-NPs, PB-UMN-CS-ES-NPs and PB-UMN-CS-FA-ES-NPs.

After administering free PB and various nanoparticle formulations (PB-UMN-CS-NPs, PB-UMN-CS-FA-NPs, PB-UMN-CS-ES-NPs, and PB-UMN-CS-FA-ES-NPs) at a dose of 5.91 mg/kg, collected organs were processed and treated with H and E stain. The organs were examined under a microscope, and images were acquired and represented in **Fig. 7.** The H & E stained images of the healthy rats were analyzed and comparisons were done with rats treated with nanoformulations to evaluate any potential toxicity arising from the administration of various formulations. A comprehensive analysis of the H & E images from healthy rats showed the absence of pathological lesions in their vital organs. Conversely, animals administered with free PB displayed pathological in vital organs. Histopathological investigations indicate that nontargeted NPs have led to a relatively enhanced safety PB treatment. Further, the administration of PB-UMN-CS-FA-NPs, PB-UMN-CS-ES-NPs, and PB-UMN-CS-FA-ES-NPs have not exhibited any visible toxic effects on the organs, and the resulting images closely resembled those of the healthy rats 59.

### 3.10. *In vivo* breast tumor imaging by USG/PA

**Figure 8:**
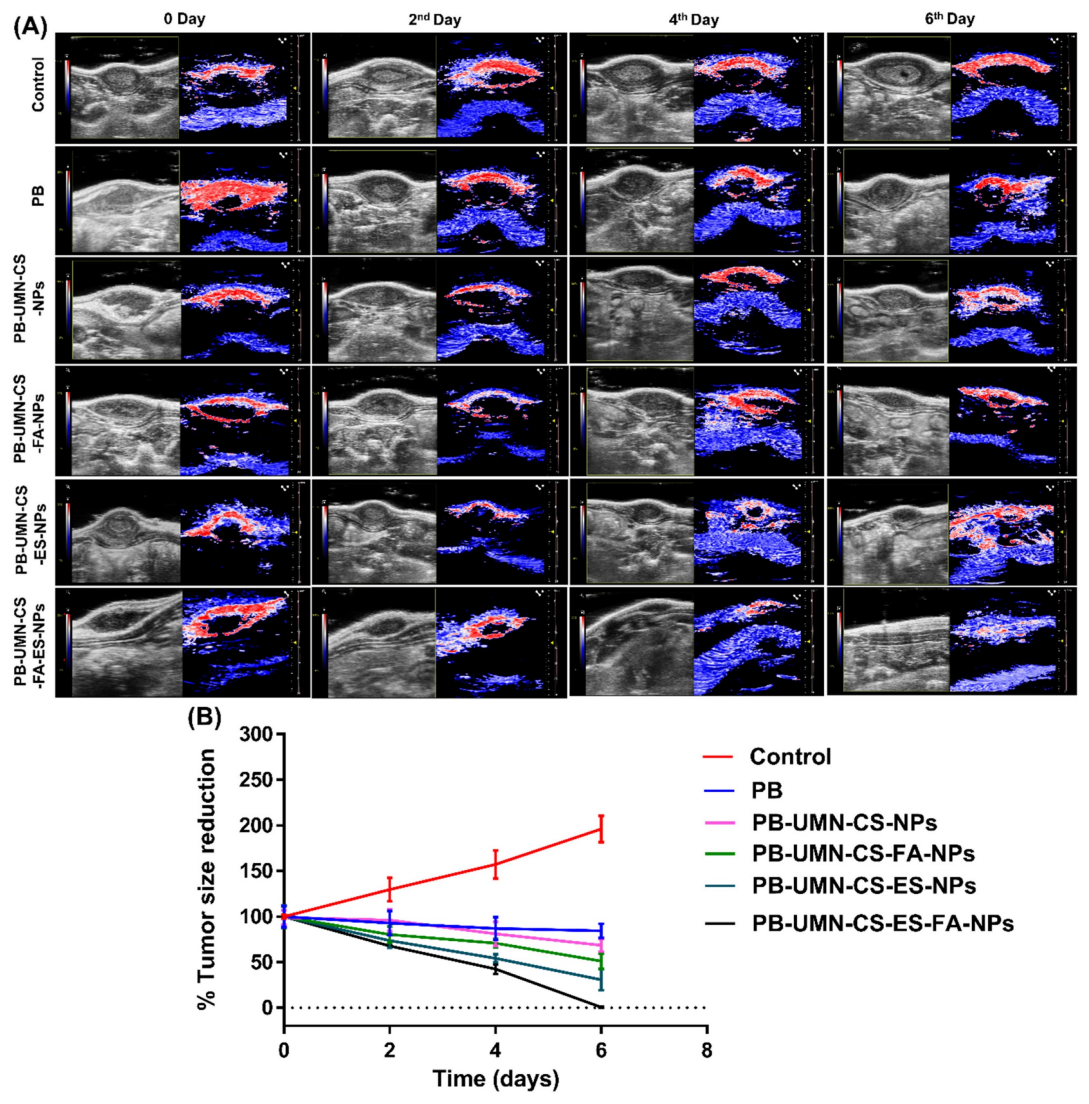
** (A)** *In vivo* breast tumor imaging of the rat by USG and PA imaging before (0 day) and after treatment (2^nd^, 4^th^ and 6^th^ days) with PB, PB-UMN-CS-NPs, PB-UMN-CS-FA-NPs, PB-UMN-CS-ES-NPs and PB-UMN-CS-FA-ES-NPs, **(B)** Comparison of breast tumor size reduction among control, PB, PB-UMN-CS-NPs, PB-UMN-CS-FA-NPs, PB-UMN-CS-ES-NPs and PB-UMN-CS-FA-ES-NPs treated groups.

This study involved the examination of SD rats with breast tumors by utilizing USG/PA imaging techniques. The imaging was conducted prior to the initiation of any treatments and afterward on the 2^nd^, 4^th^, and 6^th^ days post-treatment. The breast tumor's USG/PA pictures exhibited a notable decrease in tumor size after the injection of targeted theranostic nanoformulation, as compared to nontargeted theranostic nanoformulation and free PB. Additionally, the control group exhibited an increase in tumor size, comprising animals who were solely administered saline without any form of treatment. On the 6^th^ day of the study, it was observed that the breast tumor had totally disappeared in animals that received the dual-targeted NPs, as depicted in **Fig. 8A and 8B**.

**Figure 9:**
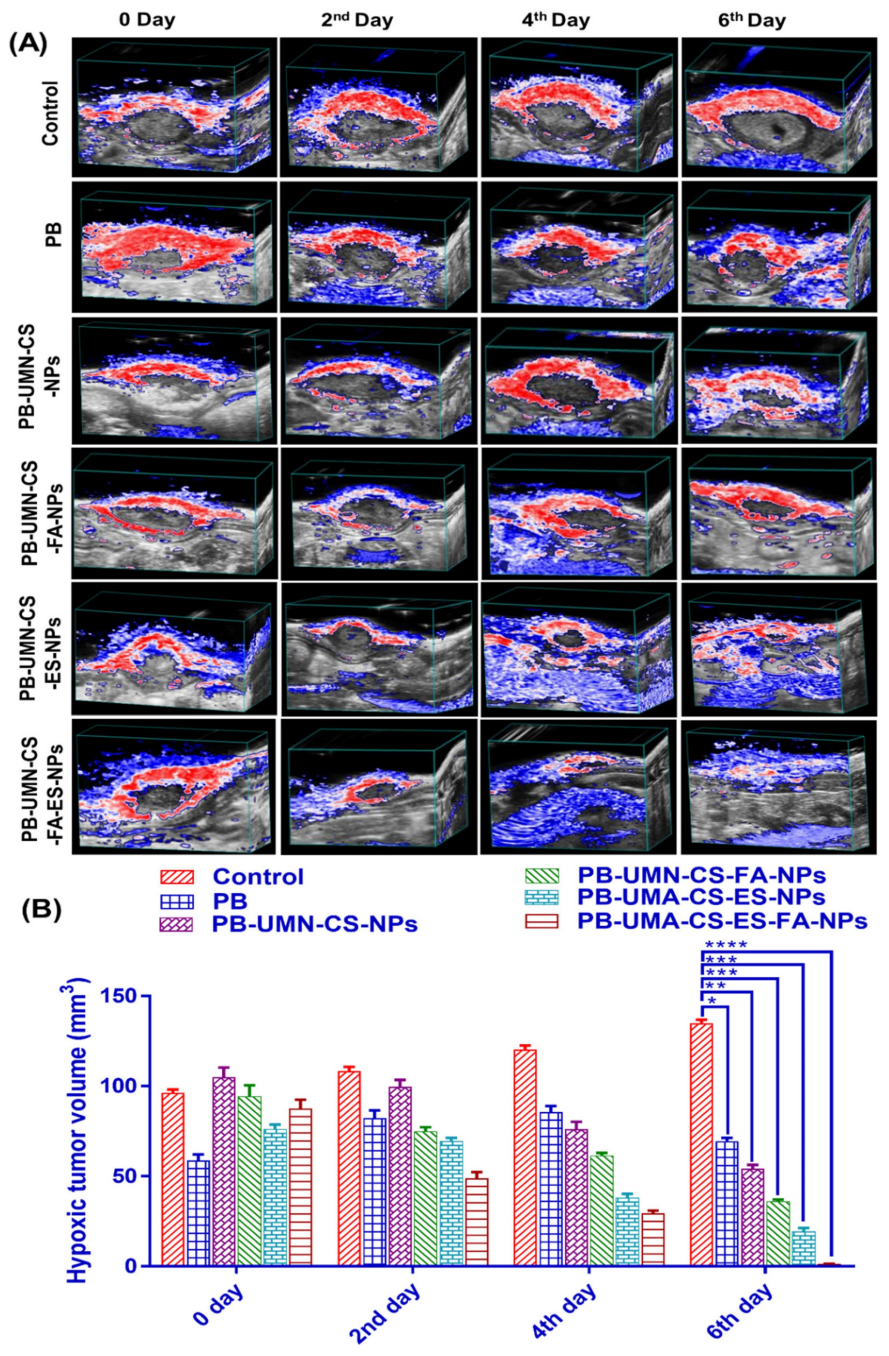
** (A)** Hypoxic breast tumor volume estimation in rats by USG and PA imaging following administration with PB, PB-UMN-CS-NPs, PB-UMN-CS-FA-NPs, PB-UMN-CS-ES-NPs and PB-UMN-CS-FA-ES-NPs and **(B)** Statistical comparison of the hypoxic tumor volume among different treatment groups. * (P < 0.05), ** (P < 0.01), *** (P< 0.001), and **** (P< 0.0001).

Tissue hypoxia is characterized by an insufficient supply of oxygen to a particular tissue, which may hinder its biological functions. Hypoxia is a well-recognized characteristic of solid malignant tumors and occurs when the oxygen supply becomes inadequate at a distance greater than 70 to 150 µm from the tumor's vascular system. This condition is caused by the rapid proliferation of malignant cells, which outpaces the development of the tumor's blood vessels, resulting in oxygen depletion in the surrounding tissue 60, 61. Experimental and clinical research have shown that hypoxia is crucial to solid tumors. The pathophysiology of malignant tumors disrupts tumor microcirculation. These abnormalities worsen oxygen diffusion, causing tumor hypoxia 62. Intratumoral hypoxia, caused by vascular endothelium functional and structural abnormalities, is often associated with a more malignant phenotype, metastasis, and treatment resistance 63. Tumors that possess similar size and structural properties might display variable levels of hypoxia and vascularization. Since, rats breast tumor were overexpressed with ER and FR, developed targeted NPs can be efficiently delivered to the breast tumor via receptor mediated transcytosis for inhibiting tumor growth and reducing hypoxic tumor volume. Before initiating the study, it was ensured that all groups of rats with breast tumors had comparable levels of hypoxic tumor volume and tumor vascularization, as represented in **Fig. 9.** Following the treatment interventions, a significant reduction (P < 0.05) in hypoxic tumor volume was observed in the group treated with NPs. Specifically, by the 6^th^ day after dual-targeted NPs treatment, hypoxic tumor volume had completely disappeared (**Fig. 9A & 9B**). However, on the 6th day free PB, PB-UMN-CS-NPs, PB-UMN-CS-FA-NPs and PB-UMN-CS-ES-NPs treated rats had 69.47 ± 1.89, 54.11±2.24, 35.77±1.24, 19.37±1.98 mm^3^ hypoxic tumor volume, respectively. It was observed that on 6^th^ day, the control group of rats had a hypoxic tumor volume of 134.95±1.27 mm^3^, which is significantly higher than the hypoxic tumor volume (96.20±1.38 mm^3^) on 0 days.

**Figure 10:**
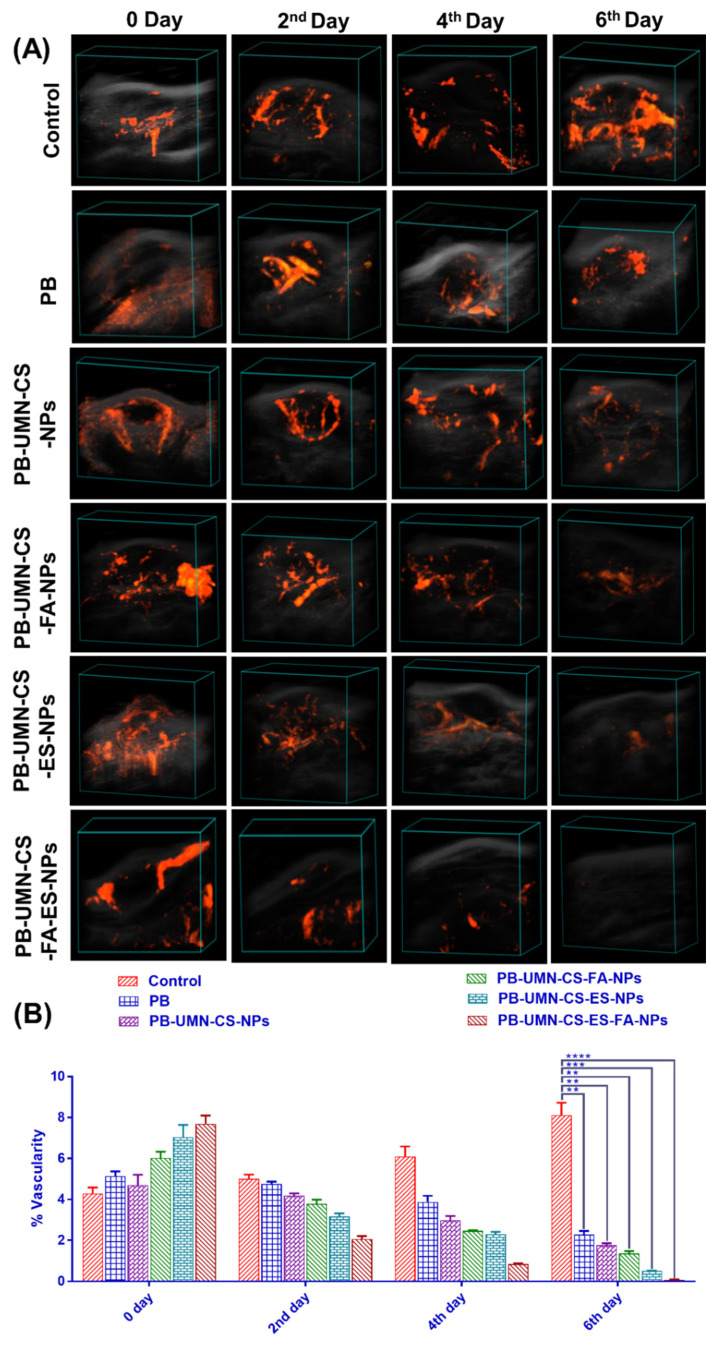
** (A)** Breast tumor images showing tumor vascularity following treatment with PB, PB-UMN-CS-NPs, PB-UMN-CS-FA-NPs, PB-UMN-CS-ES-NPs, and PB-UMN-CS-FA-ES-NPs and **(B)** Statistical comparison of tumor vascularity among different treatment groups. ** (P < 0.01), *** (P< 0.001), and **** (P< 0.0001).

Tumor growth necessitates a continuous and heightened provision of nutrients to sustain the tumor cells, thereby stimulating the formation of blood vessels (angiogenesis) both within and around the tumor. Vasculature development was arrested and progressively diminished over time, ultimately ceasing entirely by the 6th day of treatment in the rats that received dual-targeted NPs, as illustrated in **Fig. 10A & 10B**. The % vascularity on the 6^th^ day in the breast tumor of the rats after treatment with free PB, PB-UMN-CS-NPs, PB-UMN-CS-FA-NPs, PB-UMN-CS-ES-NPs, and PB-UMN-CS-FA-ES-NPs were 2.28±0.18, 1.76±0.10, 1.36±0.12, 0.51±0.08, and 0.06±0.01 %, respectively. In contrast, the group receiving FR-targeted NPs, ER-targeted NPs, and dual targeted NPs treatment exhibited a significant decline in tumor vascularity up to the 6^th^ day relative to the nontargeted and free PB group. Although, nontargeted NPs and free PB treated rats show a slight decrease in tumor vascularity, whereas the control group depicted an increase in tumor vascularity (8.12±0.60 %) over time.

Furthermore, after six days, tumor samples from the aforementioned six groups of animals were removed and subsequently underwent H & E staining. The pictures were acquired utilizing a bright microscope at a magnification of 40X. **Fig. S3A** illustrates the healthy rat breast (normal animal, without tumor), while saline treated group (consisting of breast tumor rats) received saline and displayed tumor growth in the breast lobes, which was characterized by the presence of aberrant small and big nuclei. The nuclei in each image were counted by processing H&E images into black and white (B & W) by using Image J software, as shown in **Fig. S3B**.

The number of nuclei observed in normal, saline treated, PB, PB-UMN-CS-NPs, PB-UMN-CS-FA-NPs, PB-UMN-CS-ES-NPs and PB-UMN-CS-FA-ES-NPs treated group were 62 ± 4.45, 780 ± 12.75, 650 ± 6.98, 520 ± 7.75, 390 ± 6.8, 143 ± 4.54 and 73 ± 3.15, respectively (**Fig. S3C**). An increase in the nuclei area suggests the presence of breast tumor growth. Following six days of treatment with free PB, the tumor does not exhibit many significant effects. However, animals treated with NPs formulation have demonstrated a significant reduction in the number of tumor cells (nuclei counts), and the targeted NPs treated group after 6^th^ days demonstrated a number of nuclei were nearly equal to the healthy animal group (**Fig. S3**).

**Figure 11:**
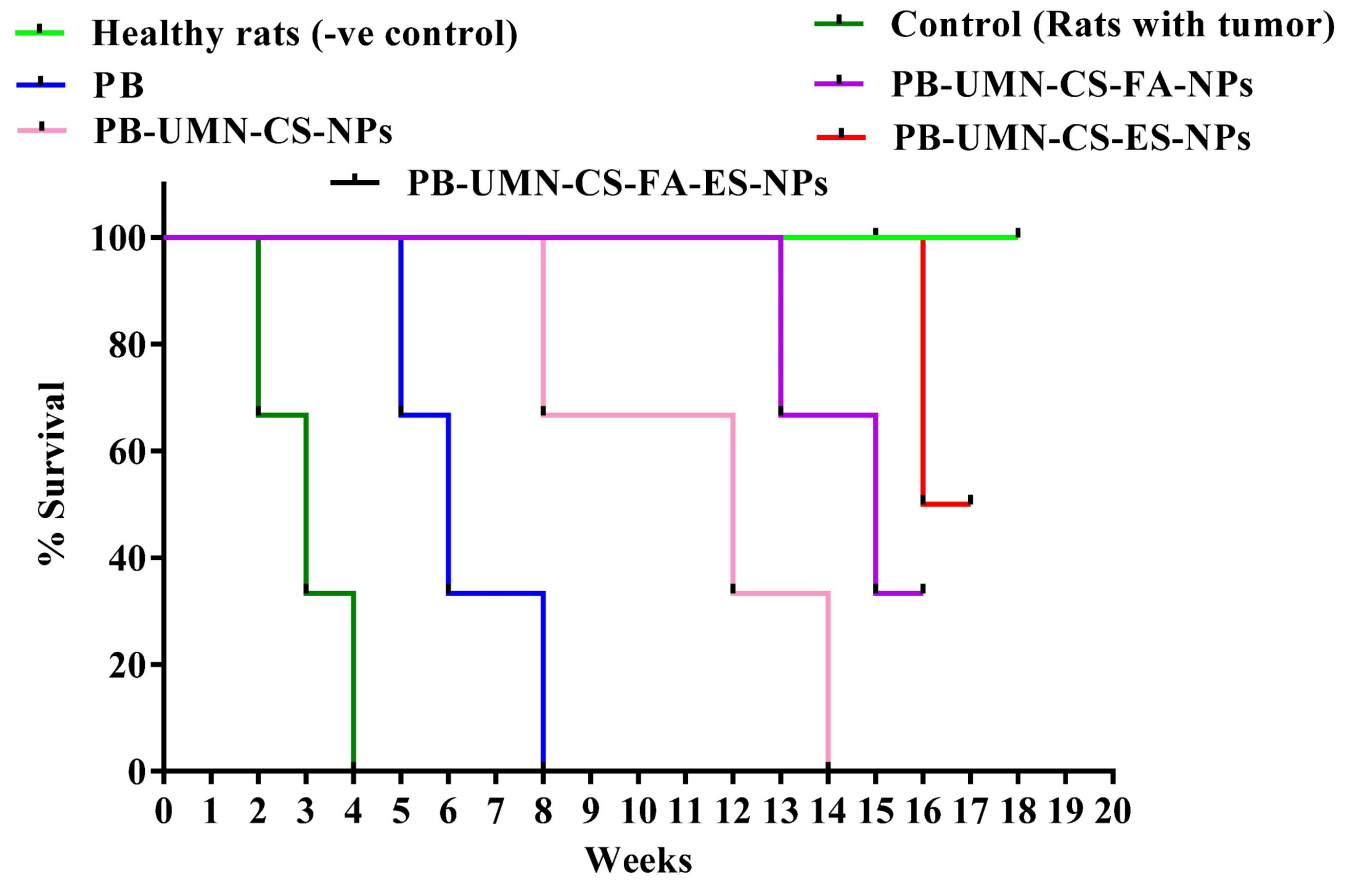
Kaplan-Meier survival analysis plot of healthy rats (without tumor), control (rats with tumor, saline treated), PB, PB-UMN-CS-NPs, PB-UMN-CS-FA-NPs, PB-UMN-CS-ES-NPs, and PB-UMN-CS-FA-ES-NPs. Healthy rats and PB-UMN-CS-FA-ES-NPs treated rats had 100 % survival (black and green lines superimposed).

**Fig. 11**. presents the post-treatment survival study conducted on rats with DMBA-induced breast tumors. The average survival rate of the animals was determined using Kaplan-Meier survival analysis throughout the 18 weeks. The administration of saline treatment enabled the tumor-bearing control rats to survive for 4 weeks, whereas the application of PB treatment extended the survival of the tumor-bearing rats to 8 weeks. Among the rats that received nontargeted NPs, they survived up to 14 weeks. FR-targeted NPs and ER-targeted NPs treated rats showed survival of 33.33 % and 66.67%, respectively, in 18 weeks. Dual-targeted NPs treatment and normal rats groups were survived completely beyond 18 weeks.

### *3.11. In vivo* fluorescent imaging of the breast tumor

**Figure 12:**
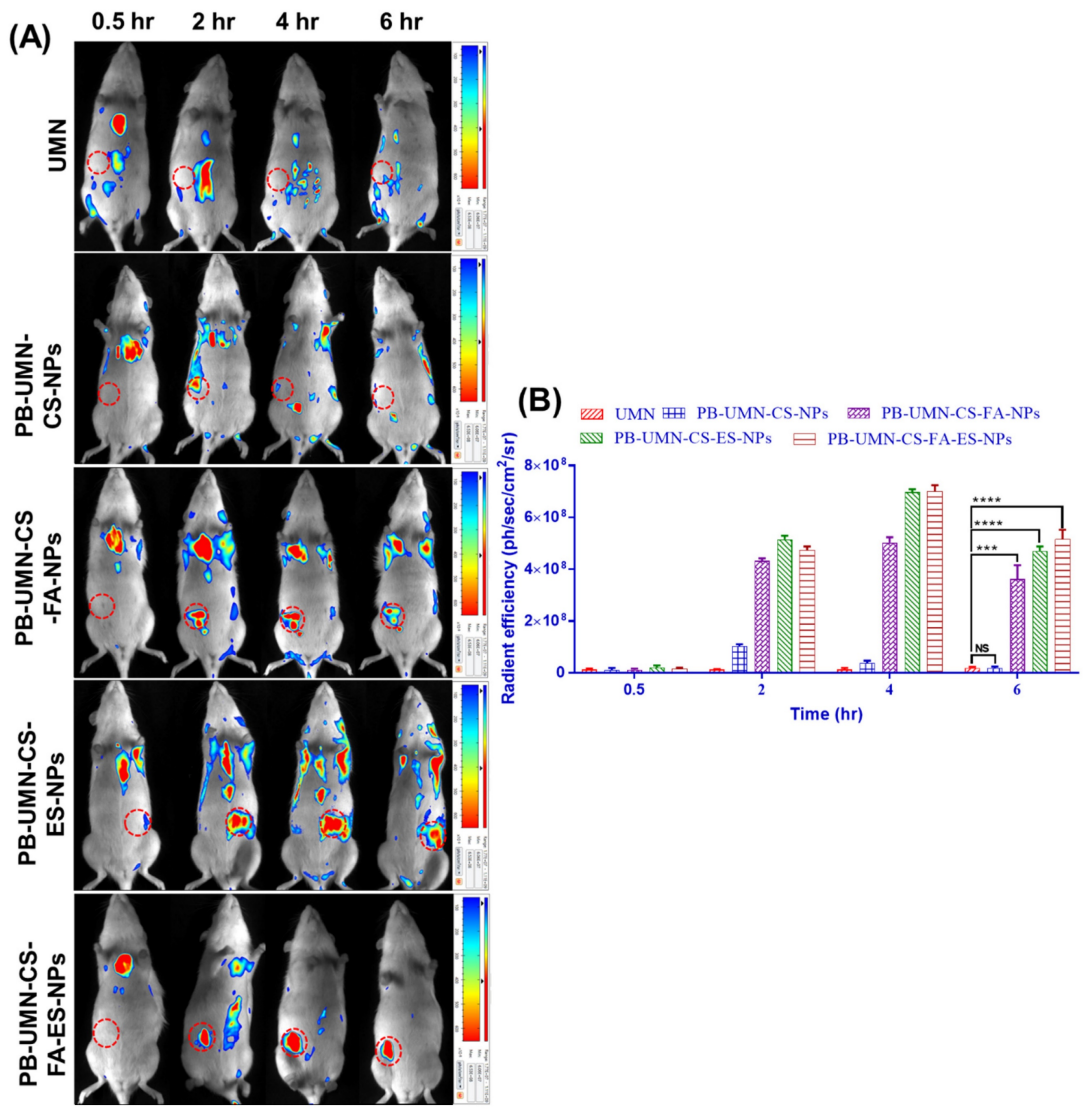
Targeted delivery of PB-UMN-CS-FA-NPs, PB-UMN-CS-ES-NPs and PB-UMN-CS-FA-ES-NPs, A) *In vivo* biodistribution of UMN, PB-UMN-CS-FA-NPs, PB-UMN-CS-ES-NPs and PB-UMN-CS-FA-ES-NPs at 0.5, 2, 4 and 6 hr after intravenous administration on DMBA induced breast cancer model. B) Histogram showing radiant efficiency of UMN, PB-UMN-CS-NPs, PB-UMN-CS-FA-NPs, PB-UMN-CS-ES-NPs and PB-UMN-CS-FA-ES-NPs after administration at different time intervals. The breast tumor has been circled (in red) on the left (first, second, third and fifth row) or right (third row) side of the breast. ns (P ≥0.05), * (P < 0.05), ** (P < 0.01), *** (P< 0.001), and **** (P< 0.0001).

The rats bearing tumors in the left or right mammary pad were selected for the study based on the size of the tumor growth. The *in vivo* breast tumor images (**Fig. 12A**) depicted the distribution pattern of free UMN (control) and prepared formulations, namely PB-UMN-CS-NPs, PB-UMN-CS-FA-NPs, PB-UMN-CS-ES-NPs, and PB-UMN-CS-FA-ES-NPs, were distributed within 0.5 hr after administration across different anatomical regions. However, after 2 hr, the targeted NPs began to accumulate specifically in the breast tumor, while the fluorescent signals from free UMN and nontargeted NPs remained low at the tumor site. Additionally, it was seen at the 6 hr time point post-administration that the specific NPs being targeted had fully aggregated at the site of the tumor. In contrast, the signals emitted by the free UMN, and nontargeted NPs were rather weak around the tumor area. Furthermore, following a duration of 6 hr, it was seen that the amount of the specific nanoformulations at the site of the tumor began to decrease, maybe as a result of metabolic processes or degradation taking place inside the microenvironment of the tumor.

**Fig. 12B** presents quantitative radiant efficiency signals from free UMN, PB-UMN-CS-NPs, PB-UMN-CS-FA-NPs, PB-UMN-CS-ES-NPs, and PB-UMN-CS-FA-ES-NPs following administration. The quantitative radiant efficiency signals after 6 hr of the administration of the free UMN, PB-UMN-CS-NPs, PB-UMN-CS-FA-NPs, PB-UMN-CS-ES-NPs, and PB-UMN-CS-FA-ES-NPs in the breast tumor region were found to be 1.87 x 10^7^ ± 0.4 x 10^7^, 1.84x10^7^± 0.6 x 10^7^, 36.2 x 10^7^ ± 5.34 x 10^7^, 47.1 x 10^7^ ± 1.75 x 10^7^ and 51.4 x 10^7^ ± 3.74 x 10^7^ ph/sec/cm^2^/sr, respectively. These findings provide conclusive evidence of the targeted delivery of PB-UMN-CS-FA-NPs, PB-UMN-CS-ES-NPs, and PB-UMN-CS-FA-ES-NPs to breast tumors. The developed theranostic formulations containing UMN have demonstrated excellent biocompatibility and *in vivo* imaging properties. Therefore, the developed formulation holds potential promise for clinical translation.

## 4. Conclusion

In conclusion, an ES and FA conjugated CS polymeric theranostic nanoformulation was developed for the targeted imaging and therapy of ER and FR overexpressed breast tumors. PB entrapment efficiencies as high as 76.82 % were observed, demonstrating that the ionic gelation and solvent evaporation process was effective in formulating the NPs. Furthermore, NPs were found to be spherical and to have smooth surfaces using FE-SEM, AFM, and TEM investigations. The XPS analysis provided conclusive evidence that ES and or FA was present on the surfaces of NPs and *in vitro* drug release study demonstrated that NPs possessed a sustained drug release profile. In addition, the analysis of cellular uptake revealed that the targeted NPs accumulated efficiently in MCF-7 cells. Notably, the free ES/FA pretreatment of cells decreased the cellular uptake of the theranostics nanoformulations because of ER and FR blocking, further confirming that receptor-mediated endocytosis is the mechanism underlying the uptake of these targeted NPs. The results of the *in vitro* cytotoxicity assay performed on T-47D and MCF-7 cells demonstrated that the dual-targeted NPs showed notably higher cytotoxicity than free PB. Specifically, there was a 54.17-fold increase in cytotoxicity observed in MCF-7 cells, while T-47D cells exhibited a 42.23-fold increase in cytotoxicity when exposed to the dual-targeted NPs*.* The dual-targeted NPs showed a remarkable capability to totally eradicate rat breast tumors in a 6-day period of therapy. This outcome was confirmed by the findings from both ultrasound and photoacoustic imaging techniques. Additionally, the targeted NPs exhibited enhanced effectiveness in decreasing the volume of hypoxic tumors and the presence of blood vessels within the tumors, as compared to nontargeted NPs and free PB. Moreover, *in vivo* optical imaging in rats demonstrated that dual-targeted theranostic NPs, were significantly accumulated in breast tumors after 6 hr of administration compared to the free UMN, nontargeted, FR-targeted, and ER-targeted theranostic NPs. In conclusion, the prepared dual-targeted theranostic NPs show considerable capability for carrying PB to ER and FR overexpressed breast tumors.

## Supplementary Material

Supplementary figures.

## Figures and Tables

**Table 1:** The formulation composition of the FR targeted nontargeted, ER targeted, and dual receptor targeted theranostic NPs

Formulations	CS (mg)	TPGS (mg)	CS-ES (mg)	CS-FA (mg)	PB (mg)	UMN (mg)	Sod. TPP (mg)
**Blank CS-NPs**	30	20	-	-	-	-	1
**PB-UMN-CS-NPs**	30	20	-	-	3	2	1
**PB-UMN-CS-ES-NPs**	20	20	10	-	3	2	1
**PB-UMN-CS-FA-NPs**	20	20	-	10	3	2	1
**PB-UMN-CS-ES-FA-NPs**	10	20	10	10	3	2	1

**Blank CS-NPs:** Blank chitosan NPs **PB-UMN-CS-NPs:** Nontargeted theranostic NPs **PB-UMN-CS-ES-NPs:** Estrogen receptor targeted theranostic NPs **PB-UMN-CS-FA-NPs:** Folate receptor targeted theranostic NPs **PB-UMN-CS-ES-FA-NPs:** Estrogen and folate receptor targeted theranostic NPs

**Table 2:** Particle size, polydispersity, zeta potential, entrapment efficiency, and IC_50_ value of developed NPs.

Batches	PS (nm) (mean ± S.D*)	PDI (mean ± S.D*)	ZP (mV) (mean ± S.D*)	EE (%) (mean ± S.D*)	IC_50_ (µg/mL) (mean ± S.D*)	
					MCF-7	T-47D
**PB**	-	-	-	-	40.63±1.94	48.15±1.85
**Blank CS- NPs**	150.1 ± 1.83	0.210± 0.048	+20.62± 0.318	-	-	-
**PB-UMN-CS-NPs**	178.4 ± 1.21	0.230± 0.042	+19.02± 0.382	74.15 ± 1.83	4.15±0.38	5.92±0.83
**PB-UMN-CS-ES-NPs**	181.6± 1.35	0.191±0.031	+13.89±0.410	76.82± 1.84	1.29±0.05	1.65±0.06
**PB-UMN-CS-FA-NPs**	185.1± 1.33	0.212±0.014	+16.72± 0.527	75.92± 2.01	1.56±0.04	2.07±0.09
**PB-UMN-CS-ES-FA NPs**	198.2± 1.43	0.198±0.021	+15.23±0.377	73.04± 1.98	0.75±0.08	1.14±0.03

*n = 3; S.D: Standard deviation **PB-UMN-CS-NPs:** Nontargeted theranostic NPs **PB-UMN-CS-ES-NPs:** Estrogen receptor targeted theranostic NPs **PB-UMN-CS-FA-NPs:** Folate receptor targeted theranostic NPs **PB-UMN-CS-ES-FA-NPs:** Estrogen receptor and folate receptor targeted theranostic NPs
