# Supplementary material for: Chitosan nanoplatform for the co-delivery of palbociclib and ultra-small magnesium nanoclusters: dual receptor targeting, therapy and imaging
Source: Nanotheranostics. 2024 Feb 12;8(2):179–201. doi: 10.7150/ntno.94364 (PMC10911970; doi:10.7150/ntno.94364)
Supplement: Supplementary file 1 — Supplementary figures. [file ntnov08p0179s1.pdf]

Supplementary data

Chitosan nanoplatform for the co-delivery of palbociclib and ultra-small magnesium nanoclusters: dual receptor targeting, therapy and imaging

Abhishesh Kumar Mehata[1], Virendra Singh[2], Vikas[1], Prachi Srivastava[3], Biplob Koch[2], Manoj Kumar[3], Madaswamy S. Muthu[1*]

[1]Department of Pharmaceutical Engineering and Technology, IIT (Banaras Hindu University), Varanasi–221005, UP, India;

[2]Cancer Biology Laboratory, Department of Zoology Institute of Science, (Banaras Hindu University), Varanasi–221005, UP, India;

[3]Nano2Micro Material Design Lab, Chemical Engineering and Technology, IIT BHU, Varanasi-221005, UP, India

---

[*]Corresponding author:
Department of Pharmaceutical Engineering and Technology, Indian Institute of Technology (BHU), Varanasi – 221005, UP, India
Tel.: +91 9235195928; Fax: +91 542 2368428;
E-mail: msmuthu.phe@itbhu.ac.in (Madaswamy S. Muthu)

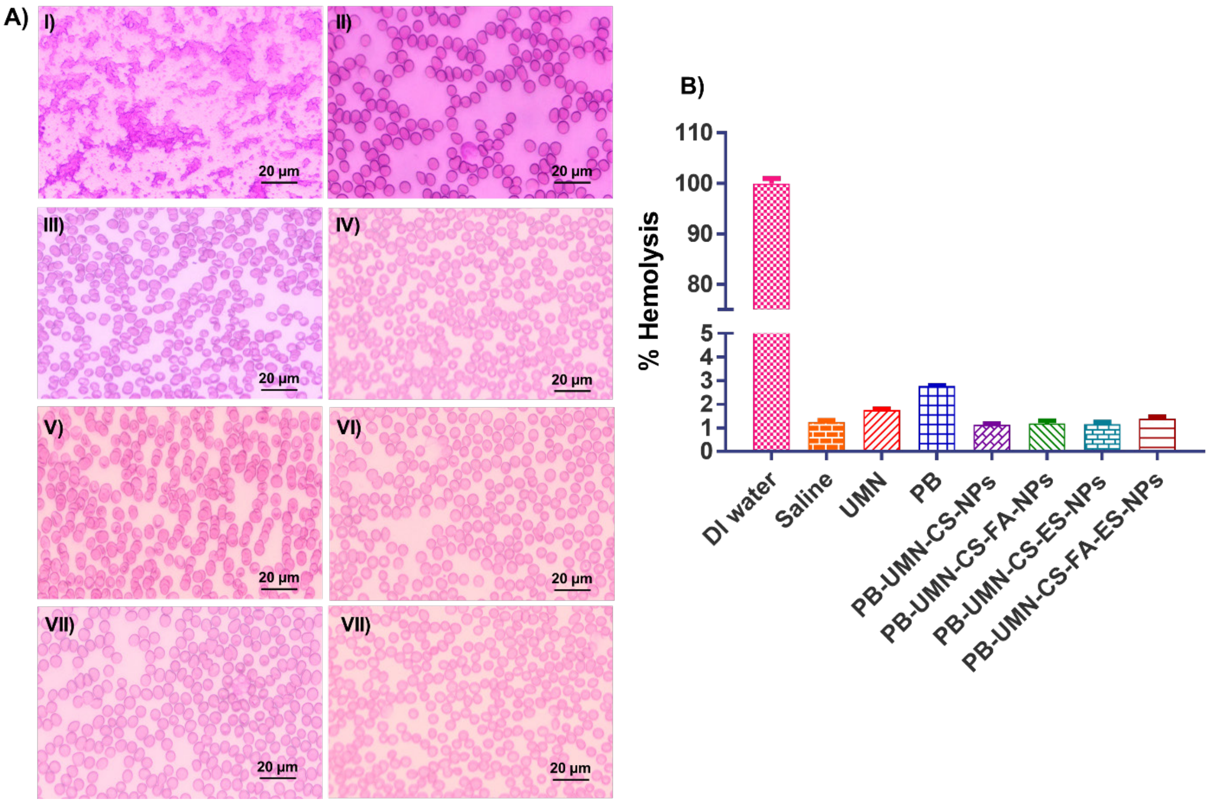

Fig. S1. (A) Hemocompatibility study; I) DI water, II) Saline, III) PB, IV) PB-UMN-CS-NPs, V) PB-UMN-CS-FA-NPs, VI) PB-UMN-CS-ES-NPs and PB-UMN-CS-FA-ES-NPs, (B) Hemolysis study; DI water, II) Saline, III) PB, IV) PB-UMN-CS-NPs, V) PB-UMN-CS-FA-NPs, VI) PB-UMN-CS-ES-NPs and PB-UMN-CS-FA-ES-NPs.

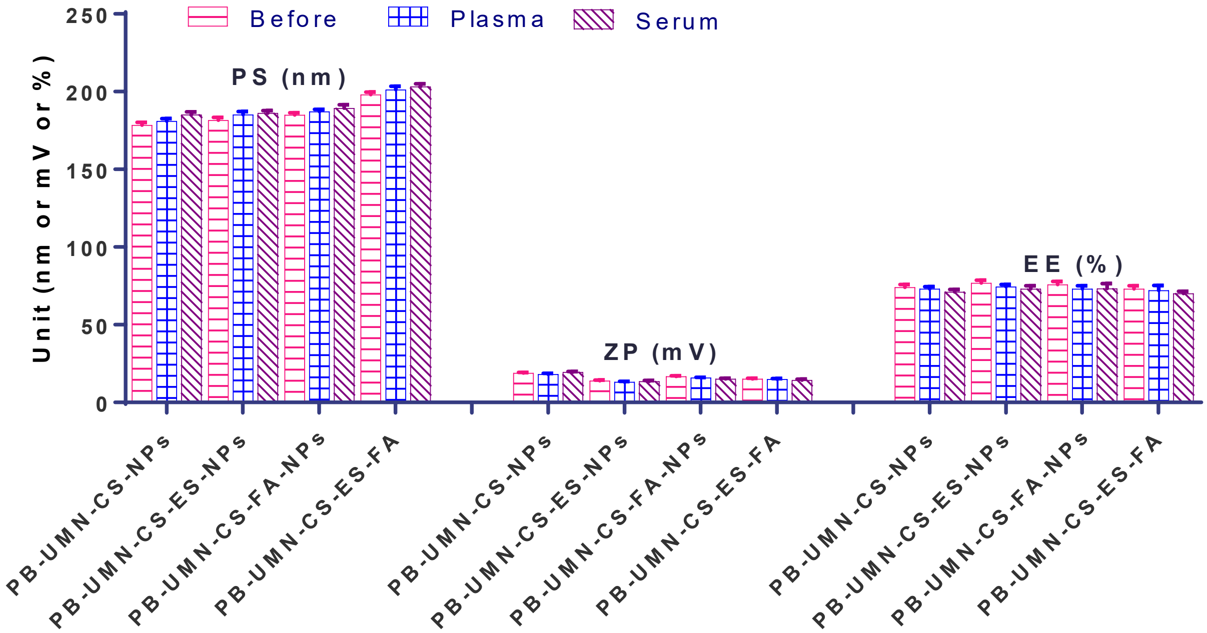

Fig. S2. *In vitro* physiological stability of the NPs in plasma and serum

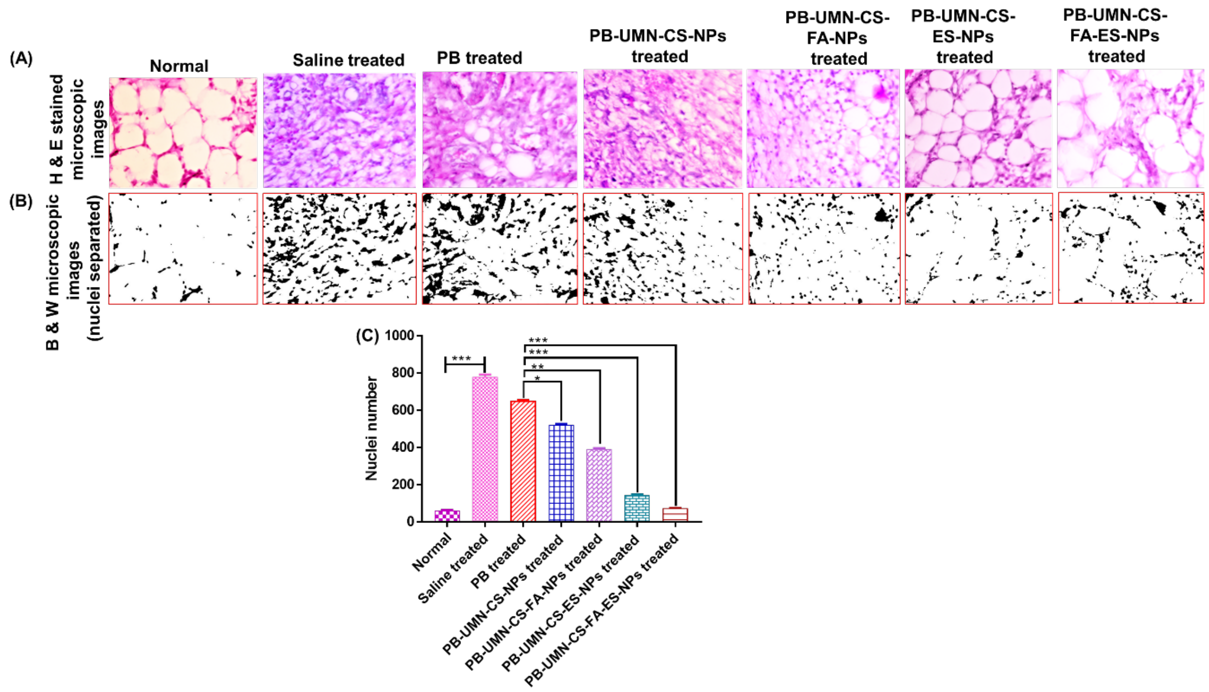

Fig. S3. A) Histopathological H & E staining of normal, saline treated, PB, PB-UMN-CS-NPs, PB-UMN-CS-FA-NPs, PB-UMN-CS-ES-NPs and PB-UMN-CS-FA-ES-NPs treated rat breast tumor samples. B) B&W images of separated nuclei of normal, saline treated, PB, PB-UMN-CS-NPs, PB-UMN-CS-FA-NPs, PB-UMN-CS-ES-NPs and PB-UMN-CS-FA-ES-NPs treated rat breast tumor sample, C) histogram showing the number of separated nuclei from HE B&W images.
